# Slowdown of Enzymatic Cellulose Conversion Emerges from Cellulase Mode of Action

**DOI:** 10.1021/acscatal.5c08098

**Published:** 2026-03-02

**Authors:** Manuel Eibinger, Gaurav Singh Kaira, Bernd Nidetzky

**Affiliations:** † Institute of Biotechnology and Biochemical Engineering, 27253 Graz University of Technology, NAWI Graz, A-8010 Graz, Austria; ‡ Austrian Centre of Industrial Biotechnology (acib), A-8010 Graz, Austria

**Keywords:** cellulose recalcitrance, enzymatic rate retardation, AFM nanomechanical mapping, hydrolysis slowdown, cellulose degradation, cellulase, cellulosome

## Abstract

The problem of early slowing of the conversion rate in cellulose hydrolysis, and the resulting large amounts of cellulase enzymes required to achieve just useful conversion efficiencies, remains a major obstacle in the development of cellulosic biofuels. While numerous studies have implicated both substrate and enzyme factors, the underlying mechanism of this rate limitation has remained unresolved. Here, using bacterial cellulose as a well-defined model substrate, we demonstrate that the reaction slowdown emerges from the specific mode of substrate degradation imposed by the cellulolytic enzyme system. We introduce nanomechanical mapping by time-lapse in situ atomic force microscopy to characterize at nanometer spatial resolution the change in surface material organization of cellulose due to enzymatic degradation. The layer-by-layer ablation of surface material utilized by fungal cellulases results in the gradual exposure of the nanomechanically stiffer (i.e., more densely organized and hence more resistant) inner core of the cellulose fibrils, which leads to a rapid decline in the conversion rate by these enzymes. Cellulases assembled into stable complexes (the cellulosome) bind almost irreversibly to cellulose. Low dynamics of their adsorption causes stalling of the cellulose degradation as the portion of unproductively bound enzymes increases during the conversion. Together, these findings reveal distinct, system-specific slowdown mechanisms and uncover a functional interplay between substrate nanomechanics and enzyme adsorption dynamics that dictates overall conversion efficiency. By disentangling these coupled rate-limiting factors, this work establishes previously unrecognized molecular design principles for engineering cellulase systems capable of overcoming the intrinsic substrate recalcitrance of crystalline cellulose.

## Graphical abstract

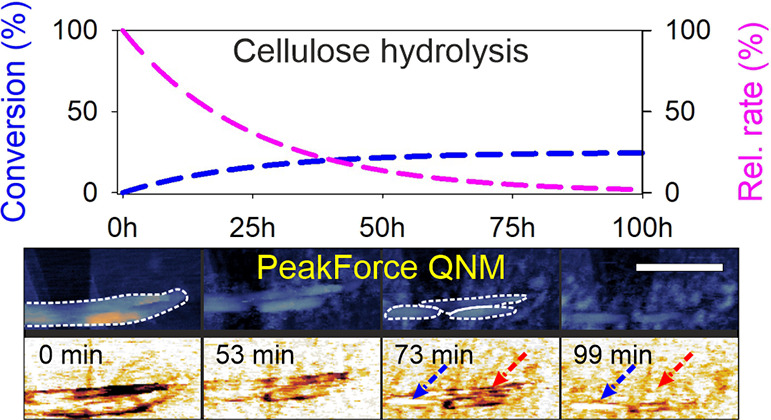

## Introduction

Advances in the sustainability of our industrialized societies require a transition to a biobased circular economy. Technologies to convert cellulosic biomass into chemical intermediates for biorefineries will be a key component of this transition.^1–5^ The term “recalcitrance” is commonly used to describe the molecular-structural features of the biomass that prevent efficient conversion of carbon from the feedstock into desired products.^6–8^ Biomass is a biopolymer composite that is approximately half cellulose. Although recalcitrance is a complex multifactor property of the biomass in relation to the particular conversion technology used,^8^ it is virtually always the difficulty in breaking down cellulose as the central component.^6^ Cellulose is a crystalline polymer composed of long linear β-1,4-d-glucose chains assembled into microfibrils a few nanometers thick.^9–11^ The microfibrils coalesce into aggregates 10–20 nm in diameter. Biochemical conversion technologies use enzymes (mostly cellulases) to selectively depolymerize cellulose into the monomer glucose.^12,13^

The recalcitrance of cellulose manifests itself in disproportionate amounts of cellulase enzymes required to achieve just useful glucose release productivity.^6,7,14^ Cellulose microfibrils are believed to be recalcitrant at the nanoscale because of their crystalline nature.^13,15^ Aggregation of the microfibrils further reduces the surface area for enzymes to attack.^16–18^ Thermochemical-mechanical pretreatment technologies target disruption of the ordered morphology of cellulose from the meso- to the nanoscale in order to facilitate the enzymatic conversion.^19–22^ However, the various preparations of crystalline cellulose (see Figure S1 and Table S1 for a representative selection) differ in their recalcitrance not primarily by the initial reactivity to cellulases, as might be expected from the different degrees of biomass processing that they represent, but by the retention of reactivity during the conversion.^8^ It follows that recalcitrance must be viewed as an emergent property of the substrate as it interacts with the cellulases during the conversion. The common feature of reaction slowdown is shown conceptually in Figure 1A and for different celluloses in Figure S1: the initial reactivity declines by ≥ 10-fold while a substantial portion (up to 70%) of the original substrate mass is still present. The “saccharification” yield, which is the common parameter to assess the conversion efficiency, is thus governed by the reaction slowdown (Figures 1A and S1).

**Figure 1:**
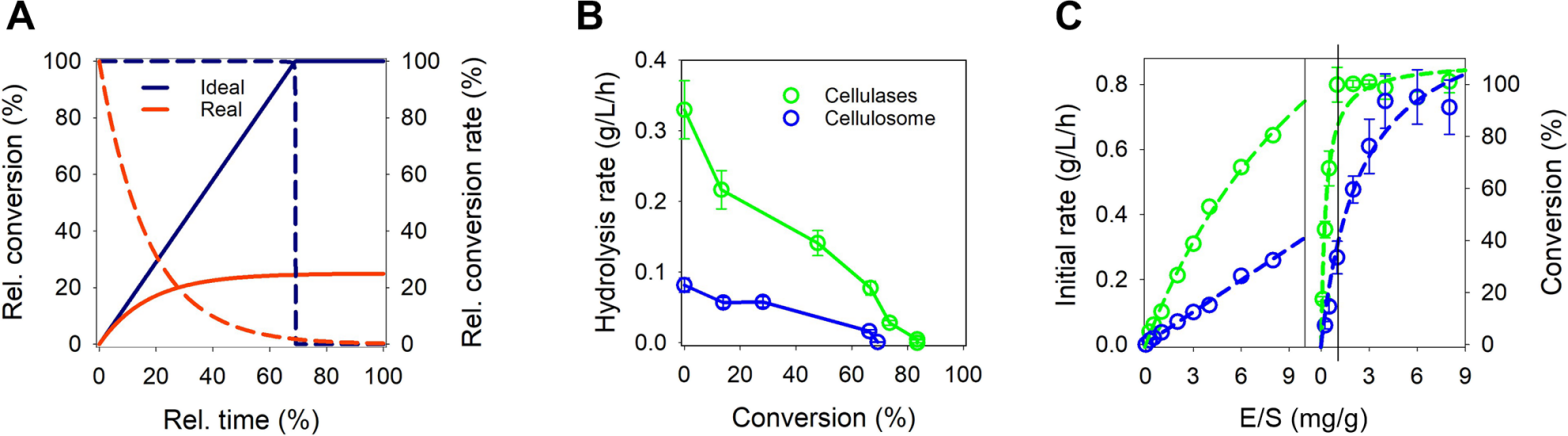
Slowdown of enzymatic cellulose conversion during hydrolysis. (A) Comparison of conversion (solid line) and rate (dashed line) for the ideal case (no slowdown) and the more realistic case (pronounced slowdown) in cellulose degradation. (B) Slowdown of bacterial cellulose conversion rates by cellulases and cellulosomes at low E/S ratios (≤2 mg/g). (C) Initial rate (within 1 h) and substrate conversion (after 24 h) at varied E/S ratio. Lines show straight-line or curve fits of the data. Error bars are SD of 3 independent experiments.

Figuring out what causes the slowdown is a subject of great fundamental interest and is central to the optimization of bioconversion technologies.^23,24^ Numerous proposals from over 5 decades of research invoke rate limitation by the cellulose substrate, the enzymes or both. Substrate-associated factors such as crystallinity,^25^ substrate surface accessibility,^26,27^ and progressively increasing recalcitrance of the material^28–31^ have been considered. However, the slowdown has also been attributed to various enzyme-associated factors, with the following considered important in particular: blocking of cellulases at nanoscale obstacles (e.g., another enzyme) at the cellulose surface;^32^ nonproductive adsorption of the cellulases;^33,34^ decline in specific enzyme activity,^35^ and deposition of inactive enzyme on the surface.^36^ Various studies propose both enzyme and substrate-associated factors in combination.^37–41^ Despite these significant efforts in research, the crucial factors underlying the enzymatic slowdown remain mechanistically unexplained.

Here, we reveal origins of the reaction slowdown from a study of the enzymatic conversion of bacterial crystalline cellulose. We introduce nanomechanical mapping by time-lapse atomic force microscopy (AFM) in a liquid environment to characterize, at nanometer spatial resolution (≤2 nm), how surface material organization in cellulose changes during the enzymatic degradation. We employ AFM force-volume methodology to obtain arrays of spatially resolved force–distance curves (FDCs) and from each FDC the height and the elastic modulus are extracted as parameters of local property of substrate surface. Total time (≈120 min) and temporal resolution (≈5 min) of recording FDC array data are carefully aligned to the evolution of the slowdown effect in the degradation process. Studies of the dynamics of enzyme adsorption to cellulose and assessment of the reactivity of the adsorbed enzyme over time are used to complement the results of nanomechanical mapping. We analyze the two main types of cellulolytic enzymes in nature: cellulases, which constitute an ensemble of individual cellulose chain-cleaving enzymes,^13,42^ and the cellulosome, which represents a stable complex of multiple cellulolytic enzyme subunits assembled on a scaffold protein (Figure S2).^43–46^ For both enzyme systems, we correlate changes in the cellulose surface nanostructure with the slowdown effect during substrate degradation. Our results demonstrate that the mechanistic origin of the slowdown differs between cellulases and the cellulosome and is tightly connected to the mode of action used by each enzyme system for the nanoscale deconstruction of crystalline cellulose.

## Results and Discussion

### Cellulose Substrate and Cellulolytic Enzymes Used

Bacterial cellulose was produced by *Komagataeibacter xylinus* (formerly *Acetobacter xylinum* was used. The substrate is a loosely organized three-dimensional network of aggregates of cellulose microfibrils (Figure S2 and S3).^42^ We refer to these microfibril aggregates also as microfibril bundles or fibers. The bacterial cellulose is of high bulk crystallinity (≥90%) and its crystalline phases conform to cellulose Iα crystal structure.^42^ The microfibrils are composed of nanodomains of highly crystalline cellulose (typical dimension: 0.1–0.5 μm) that are interspersed by short regions of lower structural order (Figure S3).^42,47^ These less organized (“amorphous”) regions of cellulose have previously been observed as preferred sites for an initial attack by cellulolytic enzymes on the cellulose fibrils, both in plant- and bacterial-derived substrates.^26,42^ Recent high-resolution AFM studies on plant cellulose nanofibrils^23,26^ have provided compelling mechanistic insight into this phenomenon, demonstrating that amorphous and internally disrupted cellulose chains act as effective breakpoints by enhancing local chain mobility and accessibility for enzyme binding and initiation of hydrolysis.

As shown in Figure S3, nanodomains of higher and lower structural order alternate in a somewhat regular fashion along the fibril length. The bacterial cellulose closely resembles plant cell wall cellulose in the nanoarchitecture of the microfibril, yet differs from it in the lack of a distinct mesoscale organization (morphology) of solid material.^48–50^ In plant cellulose, the microfibril aggregates are further assembled into densely packed fibers of much larger dimension than found in the bacterial cellulose.^10,51^ The disassembled (“micro-/nano-fibrillated”) nature of the bacterial cellulose was crucial to unveil changes in microfibril nanostructure during the enzymatic conversion, unclouded by the confounding effects of mesoscale aggregation, and to correlate these changes with the emergence of substrate recalcitrance.

We conduct conversion studies with cellulases from the fungus *Trichoderma reesei* and the cellulosome from the bacterium *Clostridium thermocellum*. Both enzymatic systems have been well characterized and serve as key models of their respective cellulase types in mechanistic and applied research.^13,43^ *T. reesei* cellulases represent an ensemble of discrete free enzymes (herein also referred to as dispersed cellulases).^13^ The *C. thermocellum* cellulosome represents multiple enzymes organized into in a stable protein complex.^43^

### Dynamic Characteristics of Cellulose Adsorption by the Cellulases and the Cellulosome, and Their Importance for the Kinetics of Enzymatic Hydrolysis

At conditions of enzyme-to-substrate mass ratio (E/S) of ≤4.0 mg/g, almost all of the enzyme used (≥95%) was adsorbed to the cellulose. The concentration of the nonadsorbed enzyme (measured as protein in solution and analyzed with SDS polyacrylamide electrophoresis; Figure S4) was below detection under these conditions. The initial rate of sugar release was dependent linearly on E/S (Figure 1C) and based on the slope of the rate versus E/S plot, the dispersed cellulases were ∼4.5-fold more active than the cellulosome. With both enzyme systems, the reaction rate dropped almost linearly in dependence of the cellulose conversion and the substrate degradation ceased in spite of ≥20% of the original substrate mass still remaining (Figure 1B). The saccharification yield was dependent on the E/S used, substantially more so (≥8-fold) for the cellulosome than the cellulases (Figures 1C and S5). When E/S was too low (cellulosome: ≤≈3 mg/g; cellulases: ≤≈1 mg/g; see Figure 1C), restriction on the conversion could not be overcome by extending the reaction time (Figure S5), suggesting that a feature of the enzyme might be its cause. We hypothesized that the decrease in reactivity might have its origin in low dynamics of enzyme adsorption on cellulose. Single-molecule enzyme studies on the bacterial cellulose have shown previously that more than half of the adsorbed cellulases^52^ and cellulosome^45^ are bound nonproductively in terms of the substrate degradation. Other authors reached similar conclusions in single-molecule studies of isolated cellulase(s) on different cellulose substrates.^52–55^ Dynamics of adsorption could arguably be critical to recover these nonproductively bound enzymes for activity. We performed competition experiments wherein uniformly ^13^C labeled cellulose was added to enzymes adsorbed on unlabeled cellulose (Figure 2).

**Figure 2:**
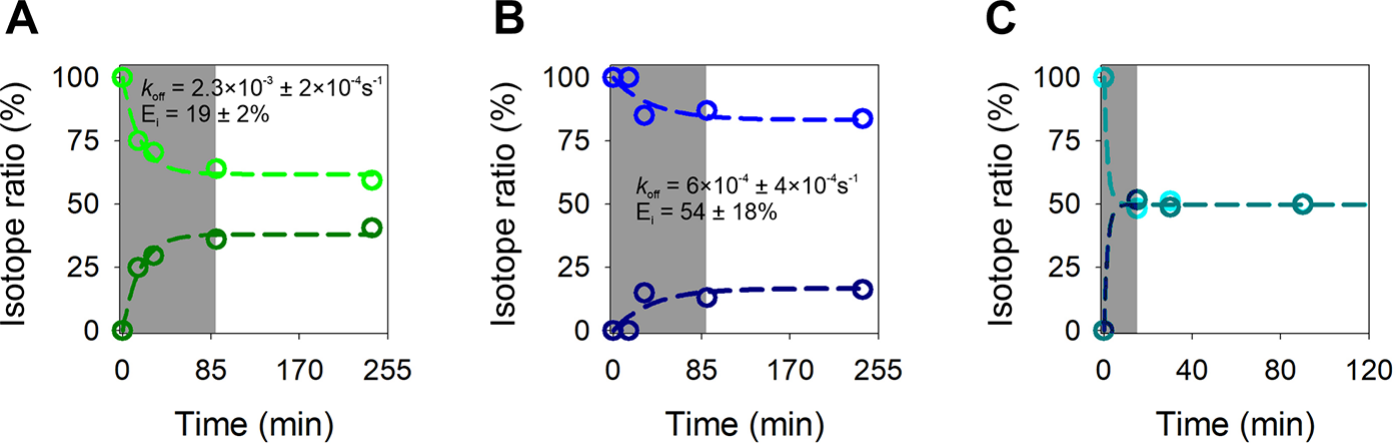
Enzyme partitioning between unlabeled and ^13^C-labeled cellulose. (A) Cellulases, (B) intact cellulosome, and (C) disassembled cellulosome. At the time of ^13^C-labeled cellulose supplementation, the enzyme concentration in solution was effectively zero due to the low enzyme-to-substrate ratio (E/S ≈ 0.83 mg/g) used. The experiment quantified how enzymes initially adsorbed on unlabeled (^12^C) bacterial cellulose redistributed between this cellulose and uniformly ^13^C-labeled cellulose, which was added at *t* = 0 min at equal concentration (1.0 mg/mL). The isotope composition of the glucose released was measured by mass spectrometry and is expressed as percentage of total glucose (^12^C, light color; ^13^C, dark color). Dashed lines represent fits of the kinetic model (Supporting Information, Equations S1–S9) to the experimental data over the time interval indicated by the gray-shaded area. The desorption rate constant (*k*_off_) and the fraction of irreversibly adsorbed enzyme (E_i_) were obtained from the model. For E_i_ = 0 (i.e., fully reversible adsorption), the ^12^C/^13^C isotope ratio approaches unity, as observed in panel C. Disassembly of the cellulosome into its enzymatic subunits was carried out following a reported protocol.^56^ Error bars represent the 10% confidence interval. No kinetic fit is shown for the disassembled cellulosome, as equilibrium distribution was already reached at the first time point.

The method is adapted from Väljamäe and co-workers who used radiolabeled cellulose to analyze cellulase desorption.^57,58^ In our experiments, the enzyme in solution was effectively zero due to the low E/S ratio used (≈0.83 mg/g or 18 nmol/g). Note that the E/S ratio was far below the maximum productive binding capacity of the major cellulase Cel7A (≈122 nmol/g) on bacterial cellulose.^59^ The ^13^C/^12^C isotopic ratio of the glucose released into solution indicates the distribution of adsorbed enzymes between ^13^C- and ^12^C-cellulose. With both cellulases and cellulosome, the evolution of isotope ratio was inconsistent with a fully reversible adsorption on the cellulose, requiring that the ratio become unity once equilibrium distribution between the two substrates has been reached.

From fit of a kinetic model to the data (see the Supporting Information, Equations S1–S9), we determined the portion of irreversibly adsorbed enzyme. This portion was higher considerably in the cellulosome (≈54%) than the cellulases (19%) (Figure 2). We show that irreversibility of cellulosome adsorption has its origin in the quaternary assembly of the enzymatic subunits on the scaffold protein. Once disassembled from the cellulosome protein complex, the enzymatic subunits exhibited full reversibility of their adsorption (Figure 2C). For both cellulases and cellulosome, the reversible adsorption was rendered tight by a very slow dissociation rate (*k*_off_; see Figure 2). Slow dissociation from cellulose is a known feature of the dispersed cellulases^24,60–62^ and the system-level *k*_off_ obtained here is consistent with earlier studies of single enzymes.^61^ We note that the irreversible adsorption can become causative of the reaction slowdown when the productively absorbed enzymes gradually change to nonproductive with time or conversion, and adsorption dynamics is too low to maintain the productive/nonproductive population balance in the absorbed enzyme via desorption and productive readsorption. The mechanistic reason for adsorbed enzyme changing to a nonproductive state emerged here from a study of the change in surface nanomechanics of the cellulose substrate while being degraded by the cellulases or the cellulosome.

### Nanomechanical Mapping of Cellulose Surface during Enzymatic Degradation

We developed a novel method of AFM-based FDC analysis to map temporal changes in the nanomechanical properties of the cellulose surface resulting from the enzyme attack. Nanomechanical mapping has been applied in previous research to characterize the surface properties of cellulose and cellulose-containing polymer blends, demonstrating its utility as a sensitive probe of polymer chain organization down to the nanoscale.^63–65^ Our measurements were performed using the PeakForce Quantitative Nanomechanics (QNM) tool implemented in the hard- and software of the Bruker AFM Dimension Fast Scan Bio instrument used. PeakForce QNM is based on force-volume methodology of nanomechanical force spectroscopy and was used here to record FDC data (Figure S6) spatially resolved at ≈2 nm resolution. The previous study of Lambert et al.^66^ has used PeakForce QNM to monitor enzymatic degradation of lignocellulosic films, however, at a much more global level of substrate morphology (μm range). The FDC data were processed to obtain local values of the elastic (Young’s) modulus *E* and the height of the cellulose surface (Supporting Information, Section 2.11–2.20). The *E* modulus presents a direct probe of the polymer organization at the cellulose surface, where higher *E* reflects increased degree of molecular order and lowered hydration of the surface chains.^63–65^ Height change was correspondingly used to monitor cellulose degradation.

The FDC analysis was performed on single fibers of the bacterial cellulose adsorbed on the surface of highly oriented pyrolytic graphite (HOPG; Figure 3). The substrate preparation for AFM analysis was described in our previous study^42^ and additional details are found in the Supporting Information (Section 2.11). The Peakforce QNM measurements were carefully optimized to enable continuous monitoring of *E* modulus and height in liquid environment at low maximum force (typically ≈2 nN) applied to the solid surface (*see* Supporting Information, Sections 2.11–2.20, Figures S6–S12). We confirmed in control experiments without the enzyme that the peak force load used did not alter the cellulose nanostructure through events of deformation or removal of material (not shown).

**Figure 3:**
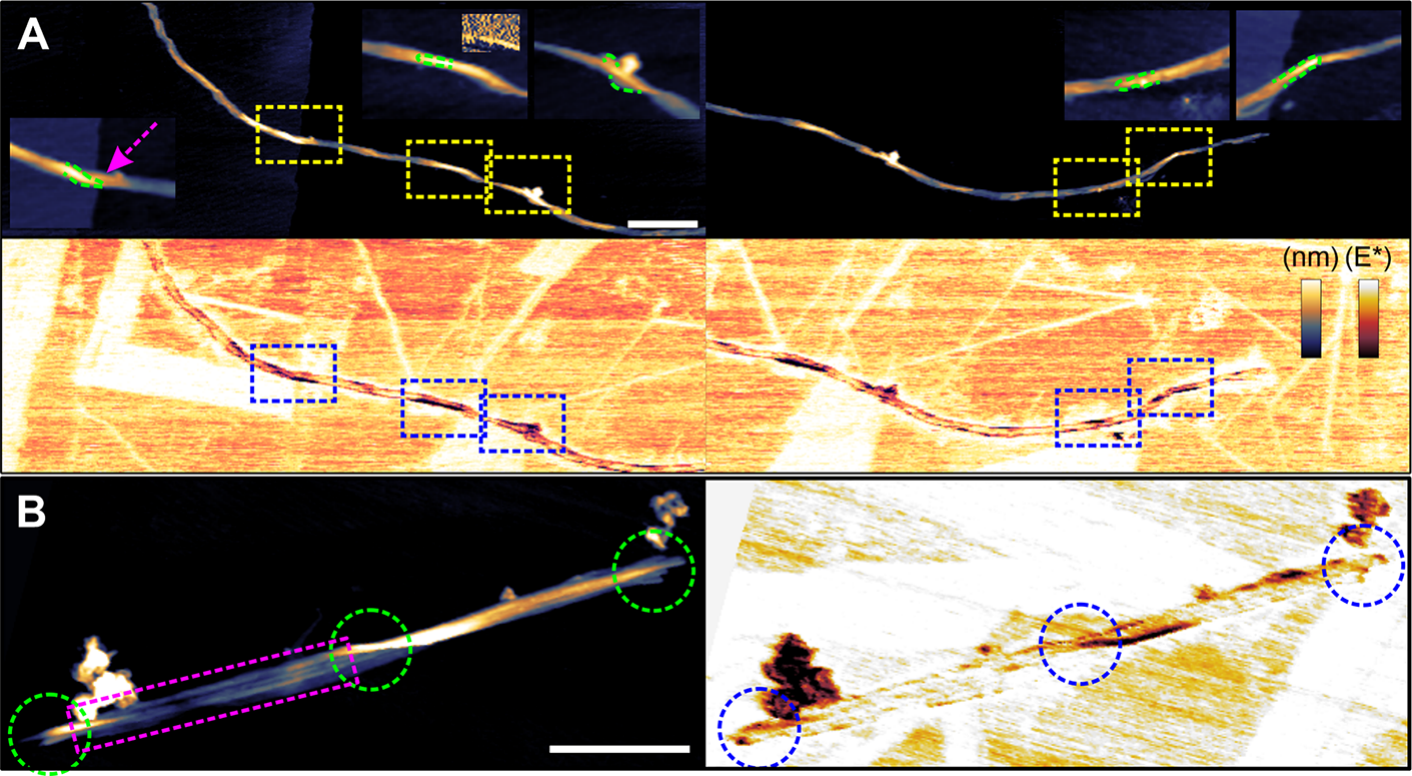
Height and *E* modulus images of a bacterial cellulose fiber. (A) Softer segments appear at regular intervals along the fiber (highlighted in squares), typically corresponding to structural features such as twists, fiber ends, or bends (see Figure S2). The insets in the height channel provide a zoomed view of the highlighted regions. (B) Short fiber composed of two elementary units (one framed in magenta), showing that both the twist region and fiber ends (highlighted in circles) exhibit a reduced modulus, consistent with the hypothesis that structural variations influence mechanical properties. Scale bar: 200 nm. False-color scales are included for the AFM images. Height range is 20 nm. The modulus covers the HOPG peak and includes 50% of the lower tail plus 10% of the upper tail.

Study of Ciesielski et al.^19^ on the nanomechanical properties of crystalline cellulose from *Cladophora* sp. algae has shown that nanoscale defects of the cellulose chain organization (e.g., breaks or bends of the microfibrils) were introduced only at ∼50-fold higher force load than was applied here. The AFM data were collected from single, initially largely intact cellulose fibers that showed regular fibrillary nanostructure over a length of ∼0.25 μm or greater (Figure 3). Fibers exhibiting structural deformations on a larger scale, like kink defects of the whole fiber or unwound regions where fibrils are dislocated from the bundle composite, were not included in the analysis. Results were obtained from time-lapse scans of a selected area of about 1 × 1 μm with a digital resolution of 4 × 4 pixel/nm^2^.

The fiber degradation was monitored over ∼1.5 h with a suitable temporal resolution of 1 scan every 5–10 min. Our earlier works^42,45,67^ have shown that the overall deconstruction of the cellulose fiber by the enzymes is reducible to multiple events of degradation on a smaller scale (≤50 nm), happening at the level of the individual microfibrils. Within a single microfibril, the regular nanodomains of high organizational order of the polymer chains^26,47,68^ were identified to represent “elementary units” of cellulose nanostructure for the enzymatic degradation. The degradation events within the single unit were previously found to be somewhat repetitive over multiple such units present along the length of the cellulose fibril analyzed.^15,26,69^ For the analysis of *E* modulus change and height loss due to degradation, therefore, the AFM data were subdivided according to the individual nanodomains of crystalline cellulose under enzymatic attack. The minimum surface area used for assessing the degradation was set to ≈10,000 nm^2^ (2500 data points). The choice of minimum area was supported from control analyses that were performed on larger individual nanodomains as a whole and by splitting the nanodomain into multiple parts of minimum size, consistent results were obtained (Figure S11).

### Analysis of the FDC Data

Our AFM measurements provided time series of spatially resolved maps of the elastic modulus *E* and height for the analyzed cellulose fibers (Figure S8). Both *E* and height were referenced to the HOPG surface (Figures S9–S10), whose modulus was assumed to remain constant throughout the incubation period and unaffected by enzyme exposure. Referencing to HOPG thus allowed correction for tip-related effects and occasional laser drift in the FDC data recorded over time. We refer to the corrected modulus as E*.

Within individual nanodomains of crystalline cellulose, E* values did not display any systematic spatial pattern across the fiber surface. Twisted regions of the fiber exhibited locally reduced E* values (Figure 3A,B). Variation of E* within each crystalline nanodomain however seemed to be predominantly statistical in nature (Figure S11).

The distribution of E* was unimodal, with a peak typically between 10 and 20 MPa (Figures S10−S12, Table S2) and a tail extending toward lower values (). This leftward skew was primarily attributed to less organized surface regions located at short terminal regions of reduced structural order (Figure 3), which recurrently appear in the image analysis due to sample drift and residual artifacts from drift correction (see Supporting Information, Section 2.18).

The mean E* value of the bacterial cellulose was consistent with previously reported data^70,71^ on the spatially averaged elastic modulus of cellulose from various sources in the hydrated state. Importantly, it was shown^72^ that the measured E* reflects the intrinsic nanomechanical properties of the cellulose surface itself, without contribution from an overlying hydration layer. Here, the applied peak force (≈2 nN) ensures direct surface contact and indentation, as demonstrated in Figure S6.^72^ The buffer did not alter the measured modulus, as the cellulose samples were never dried, stored in water, and equilibrated in buffer prior to measurement.

AFM height data (Figure 4) corroborate results of our earlier works^42,73^ showing that dispersed cellulases differ from the cellulosome in the nanoscale mode of fiber degradation used: Cellulases peel off the surface material in the lateral direction to cause thinning and shortening of crystalline nanodomains within the cellulose fibril bundle (Figure 4A,B, and Figures S13–S15, Movies S1–S3). The cellulosome produces nicked areas of cellulose surface to eventually generate fiber fragments (Figures 4C,D, S16, and S17, Movies S4 and S5). The interesting question therefore was how such different degradation modes are reflected in the nanomechanical response of the substrate in relation to the slowdown of the conversion.

**Figure 4:**
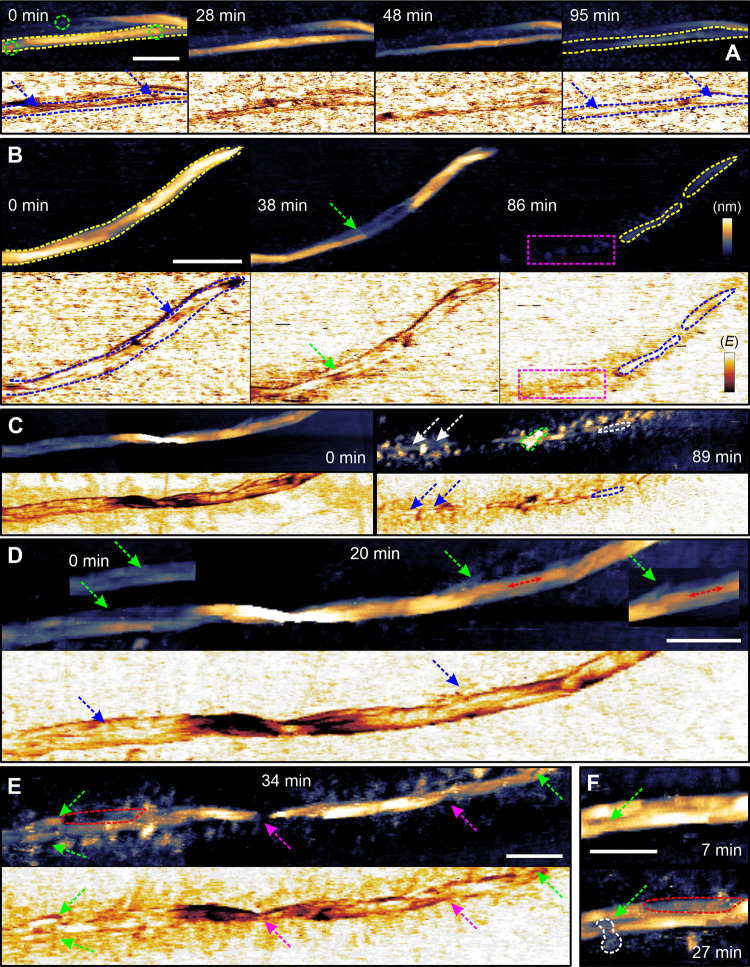
Time-lapse AFM visualization of cellulose nanostructure and nanomechanical evolution during enzymatic degradation. (A, B) AFM height images of a single cellulose fibril bundle (elementary unit, framed in yellow) degraded by cellulases. Exemplary cellulases are highlighted in green circles. The degradation proceeds through lateral peeling and thinning of the bundle into individual fibrils. Partially degraded fibrils exhibit increased relative stiffness (framed in blue). Regions of interest are marked with arrows. Irregular residual structures, such as enzymes or detached cellulose fragments, are shown in magenta and were excluded from quantitative analysis (see Figure S8). Additional images corresponding to panels A and B are provided in Figures S13 and S14, respectively. (C) AFM height image of a cellulose fiber containing two partially degraded elementary units attacked by the cellulosome (example framed in green). Isolated crystalline fibril cores remaining at late degradation stages are highlighted in white/blue. (D, E) Sequential AFM images showing local disruption and progressive fragmentation of cellulose under cellulosome action. Panel D represents an early stage of attack (∼20% conversion), featuring cavity formation and intial fragmentation (green and blue arrows) and cavity expansion (red double arrow). Insets in panel D depict the initial state (*t* = 0). Panel E (∼50% conversion) shows widening cavities (red frame), fragmentation (green arrows) and removal of fibril segments (magenta arrows). Further cellulosome images are shown in Figure S17. (F) Single cellulosome (white frame) bound to a fiber region under degradation. The green arrow indicates an expanding cavity, and the red frame highlights a fibril segment undergoing removal (∼50% conversion). Scale bars: 100 nm. Height ranges: 20 nm (A,C), 18 nm (B), 10 nm (D,E), 8 nm (F). The false-color scale of E* modulus was referenced to the mean stiffness of the background region, spanning from −50% to +10% relative to this mean.

Exemplary evolving E* distributions during enzymatic degradation are shown in Figure 5A,B and in more detail in Figure S18. The unimodal nature of the E* distribution was retained as the reaction proceeded; however, the peak E* shifted to higher values, and the distribution became narrower due to the gradual depletion of the low-E* tail. This shift suggests an increasing degree of polymer chain organization at the cellulose surface, while the depletion of low-E* values indicates preferential removal of less organized regions. Although both dispersed cellulases and the cellulosome exhibited similar overall trends in E* evolution with increasing conversion, the effect was more pronounced for the cellulases (Figure S18), showing a stronger temporal shift of the E* distribution.

**Figure 5:**
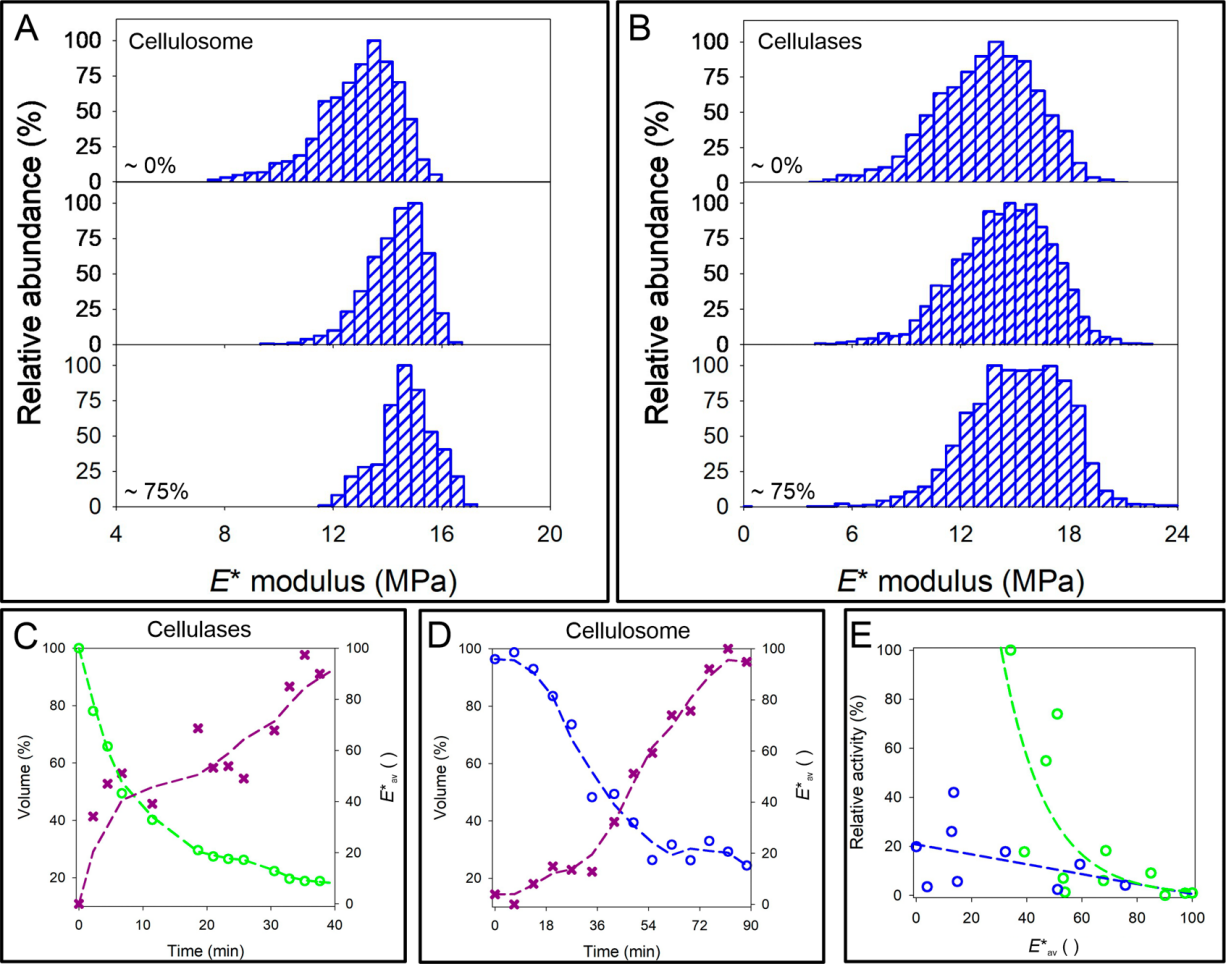
Temporal evolution of substrate nanomechanical properties during enzymatic degradation. (A, B) E* modulus of analyzed elementary units before enzyme exposure. Data in panel A were obtained from cellulosome-mediated degradation (see Figures 4C–E and S17A for representative images), while panel B shows results from cellulase reactions (Figures 4A and S14). (C, D) Temporal evolution of cellulose volume and space-averaged modulus (E*_av_), both expressed relative to their initial values, during degradation of a representative nanodomain by cellulases (C) and the cellulosome (D). Representative images corresponding to the data in panels C and D are shown in Figures S15 and S17B, respectively. (E) Comparative analysis of degradation rates for the two enzyme systems, based on the data shown in panels C and D.

To further investigate the relationship between nanomechanical properties and degradation, we calculated the spatially averaged modulus (E*_av_) as the arithmetic mean of local E* values across the analyzed cellulose nanodomains. This choice is based on the observation that the E* distributions within individual nanodomains were consistently unimodal and highly homogeneous, indicating that small local deviations are best interpreted as statistical noise rather than distinct mechanical populations. Moreover, any sub-nanometer irregularities, such as transiently detaching or enzymatically cleaved cellulose chains (Figure 3A), would act as localized sites of enzymatic activity but remain below the spatial resolution of the AFM tip (nominal radius ≈ 5 nm). Consequently, the measured E* values inherently represent an averaged mechanical response over several adjacent chains, making the mean modulus (E*_av_) the most robust descriptor of the evolving nanoscale mechanics. These E*_av_ values were then compared with cellulose volume loss, a direct measure of degradation derived from the AFM height data, as shown in Figure 4.

### Nanomechanical Properties of Cellulose and Enzymatic Degradation

Results in Figure 5D,E reveal that for both enzyme systems, E*_av_ increased as the volume loss progressed. This distinct change in E*_av_ can be attributed to the proposed hierarchical nanostructure of the cellulose fibril: the fibril core features a highly ordered organization of cellulose chains, while disorder/mobility increases toward the fibril surfaces.^10,74–76^ Although the precise nanostructure of the cellulose microfibril remains a topic of debate,^10,77,78^ the model describing a layered architecture—with a “crystalline core” surrounded by a “less densely arrayed surface shell”—is strongly supported.^79–82^

Interestingly, the temporal characteristics of E*_av_ gain, and its relationship with degradation, were fundamentally different for cellulases and the cellulosome. In the cellulase reaction, E*_av_ value increased continuously in parallel with the progression of degradation (Figures 5C and S19A). The specific pattern of E*_av_ change can be interpreted as resulting from the layer-by-layer surface degradation mode characteristic of cellulase action.

In contrast, the evolution of E*_av_ in the cellulosome reaction followed a biphasic pattern: after a short initial lag with only minor changes in modulus, a sharp increase in E*_av_ ensued (Figures 5D and S19B). Most of the fiber degradation occurred during this phase of substantial modulus increase, although some degradation also took place during the initial, constant-modulus phase. Spatially resolved analysis of AFM height data revealed that degradation without E*_av_ change was associated with cavity formation, cavity widening, and fiber fragmentation (Figures 4D–F and S16). At later stages of the reaction, fragmentation intensified, yielding smaller fiber fragments (≤150 nm length) that underwent near-complete degradation, which was accompanied by a concurrent increase in E*_av_ (Figures 4C and S17C).

### Impact of Change of Substrate E*_av_ on Enzyme Activity

By correlating E*_av_ with the enzyme activity (the first-order derivative of material loss with time), we uncover a crucial difference between cellulases and the cellulosome. Cellulase activity dropped dramatically as E*_av_ increased whereas activity of the cellulosome was almost unaffected by the same change of substrate nanomechanical property (Figures 5E and S19C). These results have immediate importance for understanding the emergence of cellulose recalcitrance in dependence of enzyme system used, as discussed in the next section below.

However, we also noted the apparent discrepancy of these findings with the biochemical results reported before (Figure S5B) that had revealed substantial decrease in cellulosome activity during the reaction. We clarify that activity retention in the cellulosome was dependent strongly on the enzyme concentration used and show that the effect appeared to be linked to the cellulosome property to become irreversibly adsorbed on cellulose (Figure 2B).

The experiment performed at elevated E/S ratio (8.9–12 mg/g), so that enzyme in solution never dropped to zero (Figure S20), confirmed the AFM results: Activity of the adsorbed cellulosome was constant during the conversion while that of the adsorbed cellulases decreased strongly (≥20-fold; Figure 6A). Activity loss in the cellulases was due to the transformative effect of enzymatic degradation on the substrate, as demonstrated in an identical experiment that used partially degraded instead of fresh cellulose (Figure 6B upper panel and Figure S21) and in excellent agreement with the AFM evidence on cellulases shown before (Figures 4A, 5A,C, S14, and S15).

**Figure 6:**
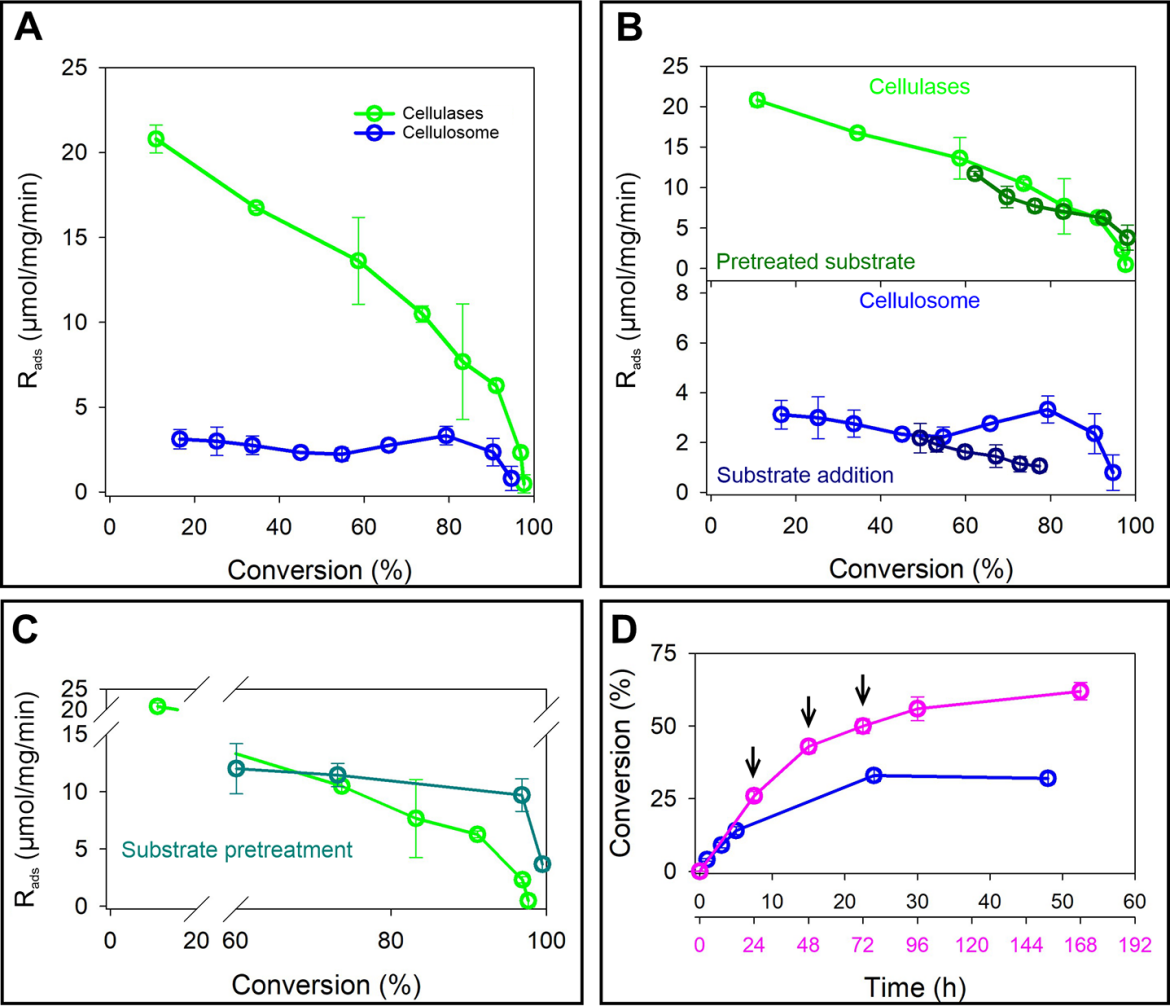
Biochemical experiments designed to explore the origin of cellulose recalcitrance. R_ads_ is the specific glucose release rate of the adsorbed enzyme. (A) R_ads_ evolution during substrate conversion by cellulases (E/S: 8.9 mg/g) and cellulosome (E/S: 12 mg/g). (B) Upper panel: R_ads_ evolution for cellulases using a partially hydrolyzed substrate (≈50% conversion by previous treatment with cellulases). R_ads_ evolution with the fresh substrate is shown for reference. Lower panel: R_ads_ evolution for the cellulosome upon the addition of fresh cellulose (1.0 g/L) at ≈45% conversion of the original substrate. The cellulose was added to deplete the free cellulosome in solution (E/S: 2.7 mg/g) by adsorption to cellulose. (C) Effect of substrate pretreatment by the cellulosome on the evolution of R_ads_ for cellulases. The untreated substrate is shown as reference. (D) Effect of substrate addition in portions (0.25 g/L; indicated by arrows) to keep the E/S ratio at 4 mg/g during the conversion, thus ensuring that there was always free cellulosome present in solution. This is contrary to the reference reaction that involved all of the substrate was added at once, so the E/S ratio was 1.0 mg/g.

Activity loss in the cellulosome must occur for a different reason, and we hypothesized that its origin could lie in the irreversibly adsorbed molecules getting trapped in an inactive state on the cellulose. Attack on substrate from the cellulosomes in solution might promote release of the trapped molecules, in order for them to return to an active state directly on the cellulose surface or via desorption and readsorption. The overall process of assisted release might be critical to reinstate the dynamics of cellulosome-cellulose interaction required for maintenance of the enzyme activity. We tested the mechanistic idea by adding fresh cellulose to a cellulosome reaction at constant activity in order to deplete the enzyme in solution by adsorption to solid substrate (Figure S22). Indeed, once the soluble enzyme was removed, the cellulosome activity started to decrease as expected (Figure 6B lower panel).

### Mechanistic Interpretation of the Reaction Slowdown

The evidence shown in this study identifies cellulose recalcitrance^6–8^ (manifested in the enzymatic reaction slowdown) as an emergent property of the microfibril-bundle nanostructure of the bacterial cellulose substrate, dependent on how the enzymes used interact with that structure during degradation. For the sake of the mechanistic interpretation, it should be kept in mind that the bacterial cellulose used here represents a special type of nanofibrillated substrate that is strongly hydrated additionally.^83,84^ Despite these structural characteristics different from most rederived cellulosic substrates,^77,85,86^ the effects observed at the single microfibril-bundle level of the bacterial cellulose (Figure 3) have general significance. We find that the cellulase mode of layer-by-layer surface ablation (Figure 4A, B; see refs 42,67 ) loses efficiency when it confronts the highly organized inner core of the cellulose microfibril. The result agrees with earlier work^87^ that showed decreased cellulase adsorption and lowered conversion rates on prehydrolyzed (“thinned”) fibers of bacterial cellulose. There are compelling reasons for why the crystalline fibril core can show enhanced resistance to cellulase attack. One is that multienzyme synergy, that between internally chain cleaving endocellulases and chain end-cleaving exocellulases in particular,^42,67^ ceases on such kind of cellulose material which is low in nanostructural surface defects that were shown previously to serve as initiator sites for endoexo cooperative activity.^67^ Another is the low number of accessible cellulose chains when the chain packing is tight and therefore the free energy of local chain decrystallization is large.^15^

From a thermodynamic perspective, this increasing resistance can be rationalized in terms of the high cohesive stabilization of crystalline cellulose, implying a substantial energetic barrier for chain extraction.^88^ However, it is important to note that while decrystallization and chain extraction require a significant local energy input,^89^ these barriers are ultimately overcome during enzymatic hydrolysis. The free energy change for the cleavage of the β-1,4-glycosidic bond is exergonic (≈−3 to −4 kcal/mol),^90^ and the high solvation energy of the resulting sugars provides a strong additional driving force. Consequently, the coupled process of decrystallization, hydrolysis, and solvation is overall energetically “downhill.” Notably, the absolute magnitudes of lattice energies^91,92^ and of the cohesive energy density (CED)^88^ are larger (by ∼1–2 orders of magnitude) than the hydrolysis energy, highlighting that the crystalline core represents a kinetic barrier rather than a thermodynamic constraint, which may explain the progressive slowdown of enzymatic digestion toward the crystallite’s interior.

Phenomenologically, the better-ordered crystallite core,^93^ as suggested by the observed increase in nanomechanical stiffness (E*), is likely even more tightly packed, further enhancing local stability. In contrast, disordered or less densely packed regions exhibit reduced cohesive stabilization, facilitating local chain mobility, solvation, and enzymatic accessibility. We emphasize, however, that these energetic considerations are intended to support the discussion conceptually and are not based on direct energetic measurements in the present study.

Lastly, the surface-dependent solvation structure of the crystalline cellulose core can arguably be different from that of the intact fibril.^94^ Processive enzymatic degradation happens along hydrophobic strips of the crystallite surface,^95,96^ resulting in an increased exposure of hydrophobic surface as the conversion proceeds.^97^ Specific solvation structures are prominent characteristics of different cellulose surfaces.^11,98^ The coupling between solvation structure and reactivity of the cellulose surface toward enzymatic degradation is not well understood,^99–104^ (for modeling works, see refs 105,106 ) but besides affecting the chain cleavage itself it can modulate enzyme access to the solid surface^105,107^ and control desorption of the cleavage products for their complete dissolution into the bulk liquid.^89^ Recent review of Westh and co-workers discusses thermodynamic limitations of depolymerization of crystalline cellulose and how cleavage product dissolution can contribute to overcome them.^62^

The cellulosome mode of fiber fragmentation (Figure 4D,E; see refs 42,45 ) averts limitations of the cellulases, yet it encounters restriction due to low dynamics of cellulose adsorption and adsorbed cellulosomes getting irreversibly trapped in nonproductive states. Tentatively, the larger size and the multisubunit structure of the cellulosome can generate effects of multivalent adsorption (with consequent changes in the surface solvation structure) and spatial proximity to promote synergy among endo- and exocellulase activities in surface degradation. It is tempting to speculate that the retention of adsorbed endocellulases is critical to maintain high specific activity.

These insights and conjectures lead to the identification of new engineering targets to overcome recalcitrance. Induction of structural defects into the fiber core, such as the ones caused by the fragmentation activity of the cellulosome, is expected to benefit the cellulases. A substrate pretreatment by the cellulosome indeed mitigated the slowdown of the cellulase rate in substantial degree and did so until the cellulose conversion was effectively complete (Figures 6C and S23). For the cellulosome, adding the substrate in portions over time according to progress of the conversion prevented depletion of the enzyme from solution and so enabled an improved saccharification yield at low E/S ratio (Figure 6D). Development of cellulases and cellulosomes optimally active at the same conditions for joint use in cellulose processing^108,109^ could thus present a promising target for molecular enzyme engineering.^110–113^

## Conclusions

Deepened mechanistic insight into the causes of the slowdown in enzymatic cellulose conversion is of great fundamental interest and has practical importance in the development of advanced bioconversion technologies. The reaction slowdown for bacterial cellulose conversion by cellulases and the cellulosome is revealed here as an emergent property of the substrate’s nanostructure, dependent on how the different enzyme systems interact with that structure during the degradation. Evidence is presented from an advanced combination of biochemical experiments and AFM-based characterization. Nanomechanical surface mapping of the substrate fiber undergoing enzymatic degradation is shown for the first time. The ablative mode of removal of surface material by the fungal cellulases promotes the gradual exposure of the nanomechanically stiffer inner core of the cellulose fibrils. This results in a rapid decline in the conversion rate by the enzymes. Possible molecular reasons for the effect are discussed. The cellulosomes bind almost irreversibly to the bacterial cellulose. Low dynamics of their adsorption causes stalling of the cellulose degradation as the portion of unproductively bound enzymes increases during the conversion. In summary, therefore, the current work reveals distinct mechanistic factors that control the conversion efficiency of cellulases and cellulosome in degrading bacterial cellulose (a type of nanofibrillated cellulose). These insights identify previously unknown engineering targets to overcome the phenomenon of “substrate recalcitrance” in bacterial cellulose and, with cautious extension, other types of celluloses.

## Methods

A summary of the experimental methods is presented here, with full methodological details, including materials, protocols, data processing workflows, and validation experiments, provided in the Supporting Information.

All chemicals were of the highest purity (Carl Roth, Germany). Enzyme systems (*Trichoderma reesei* cellulases, *Clostridium thermocellum* cellulosomes, and disassembled cellulosomes) were prepared and characterized as reported previously (Supporting Information, Section 2.1–2.3).^42^

Bacterial cellulose (BC) fibers, both unlabeled and ^13^C-labeled, were produced, processed, and stored according to established protocols (Supporting Information, Section 2.4).^42^ Enzymatic reactions were performed at defined enzyme-to-substrate ratios (E/S) under controlled temperature and agitation. β-Glucosidase was added to all reactions to convert soluble oligosaccharides to glucose. Reaction samples were taken over time, quenched, and analyzed enzymatically for glucose and protein concentration (Supporting Information, Sections 2.5 and 2.10).

Competition experiments between unlabeled and ^1^3C-labeled BC were performed to study enzyme partitioning. The fraction of enzyme adsorbed on each substrate was determined from glucose release and isotope ratio analysis using LC/Q-TOF MS. Enzyme distribution was modeled using a Langmuir-type adsorption approach (Supporting Information, Sections 2.6–2.9, eqs S1–S9), with kinetic parameters obtained from MATLAB R2023b fitting. Specific activity of adsorbed enzymes (R_ads_) was calculated as the ratio of glucose release rate to adsorbed enzyme, with careful control of substrate and enzyme loadings.

AFM measurements were performed in buffer using a Bruker Dimension FastScan Bio. BC fibers were deposited on HOPG and imaged in liquid. Probe types, spring constants, tip radii, and peak forces were optimized for each enzyme system to ensure high-resolution imaging while minimizing imaging artifacts. Elementary fiber units (∼500 nm length, 10–30 nm width) were selected for analysis, excluding fibers with major defects. Time-lapse imaging recorded topography and local Young’s modulus (*E*) for each fiber unit (Supporting Information, Sections 2.11–2.12).

Raw AFM data were processed following a structured workflow (see schematic in Figure S7) and corrected for tilt, background, and drift using Gwyddion and custom MATLAB routines.^42,114^ Fibers were identified and masked, with modulus values extracted and normalized to the HOPG background, yielding normalized stiffness (E*). Space-averaged E* per elementary unit (E*_av_) was calculated for each frame to quantify temporal changes in nanomechanical properties. Units with irregular distributions or insufficient data were excluded; heavily degraded fibers were manually masked to reduce artifacts. E*_av_ values were linearly scaled (0–100%) to enable comparison across experiments and enzyme systems (Supporting Information, Section 2.13–2.17).

Global volumetric degradation was determined from the same AFM sequences. Height images were drift-corrected and cropped to consistent regions, fiber pixels summed, and relative activity (% per min) calculated from the temporal decrease in pixel sum (SI, eq S10). This allowed direct correlation between stiffness evolution (E*_av_) and material loss (Supporting Information, Section 2.13–2.20).

## Supplementary Material













## Notes

Time lapse observation of cellulases degrading a partial elementary unit (AVI)
Cellulases at high loading degrading an elementary unit (AVI)
Time lapse observation of cellulases degrading an elementary unit at elevated temperature (AVI)
Time lapse observation of cellulosome degrading multiple elementary units of a BC fiber (AVI)
Time lapse observation of cellulosome degrading an elementary unit of a BC fiber (AVI)
Detailed description of all materials and methods; methods of the preparation, purification and characterization of unlabeled and ^13^C labeled bacterial cellulose, cellulosomes and cellulases; detailed description of hydrolysis experiments, kinetic modeling, and analytics; sample preparation for AFM observations; AFM operation, image processing, and data analysis; and additional figures, AFM images, and movies (PDF)

## Abbreviations

AFM: atomic force microscopy
HOPG: highly ordered pyrolytic graphite
SI: supporting information
CED: cohesive energy density
