# Supplementary material for: Slowdown of Enzymatic Cellulose Conversion Emerges from Cellulase Mode of Action
Source: ACS Catal. 2026 Mar 2;16(6):5603–15. doi: 10.1021/acscatal.5c08098 (PMC13010264; doi:10.1021/acscatal.5c08098)
Supplement: Supplementary file 6 [file cs5c08098_si_006.pdf]

Supporting Information for

Slowdown of Enzymatic Cellulose Conversion Emerges from Cellulase Mode of Action

Manuel Eibinger[1,#], Gaurav Singh Kaira[1,2,#], and Bernd Nidetzky[1,2,]*

[1]Institute of Biotechnology and Biochemical Engineering, Graz University of Technology, NAWI Graz, 8010 Graz, Austria

[2]Austrian Centre of Industrial Biotechnology (acib), 8010 Graz, Austria

Equal first authors

[#]M.E. and G.S.K. contributed equally

Corresponding Author

*Correspondence should be addressed to B.N. (bernd.nidetzky@tugraz.at)

1 Table of content

2 Materials and Methods S4

2.1 Cellulases........ S4

2.2 Cellulosomes S4

2.3 Disassembled cellulosomes S4

2.4 Bacterial cellulose S5

2.5 Enzymatic conversion of BC S5

2.6 Partitioning of enzymes between unlabeled and ^{13}C-labeled BC S6

2.7 Kinetic model of enzyme partitioning between unlabeled and ^{13}C-labeled cellulose S7

2.8 Data fitting........ S9

2.9 Specific hydrolysis rate of adsorbed enzyme S10

2.10 Enzymatic reactivity of partially hydrolyzed BC S10

2.11 Atomic force microscopy (AFM) – nanomechanical characterization of cellulose deconstruction........ S11

2.12 BC fibers and their elementary unit of nanostructure........ S12

2.13 AFM data structure and workflow S13

2.14 AFM data acquisition and processing for analysis of *E* modulus and derive parameters S13

2.15 Fiber masking and *E* extraction from Height and Modulus Matrices S14

2.16 Calculation of the space-averaged *E* modulus (E^*_{av})........ S14

2.17 Statistical evaluation and quality control of elementary fiber units........ S15

2.18 AFM data processing and correction of AFM time-lapse images for pixel-based calculations........ S15

2.19 AFM movie construction and .png image generation........ S16

2.20 AFM based degradation analysis S16

3 Supporting Figures and Tables........ S18

3.1 Fig. S1. .... S18

3.2 Fig. S2. .... S19

3.3 Fig. S3. .... S20

3.4 Fig. S4. .... S21

3.5 Fig. S5. .... S22

3.6 Fig. S6. .... S23

3.7 Fig. S7 .... S24

3.8 Fig. S8. .... S25

3.9 Fig. S9. .... S26

3.10 Fig. S10. .... S27
3.11 Fig. S11. .... S28
3.12 Fig. S12. .... S29
3.13 Fig. S13. .... S30
3.14 Fig. S14. .... S31
3.15 Fig. S15. .... S33
3.16 Fig. S16. .... S34
3.17 Fig. S17. .... S35
3.18 Fig. S18. .... S36
3.19 Fig. S19. .... S37
3.20 Fig. S20. .... S38
3.21 Fig. S21. .... S39
3.22 Fig. S22. .... S40
3.23 Fig. S23. .... S41
3.24 Table S1. Reaction conditions for conversion of different substrates shown in Fig. S1. .... S42
3.25 Table S2. Compilation of mean and standard deviation of exemplary E^* datasets from elementary units S43
4 Supporting Movie captions S44
4.1 Movie S1 .... S44
4.2 Movie S2 .... S44
4.3 Movie S3 .... S44
4.4 Movie S4 .... S44
4.5 Movie S5 .... S45
5 Supporting References S46

2 Materials and Methods

Unless stated, all chemicals were of the highest purity available from Carl Roth + Co KG (Karlsruhe, Germany).

2.1 Cellulases

The cellulases are from *Trichoderma reesei* (strain SVG17)[1] and were obtained as described in earlier work.[2] The preparation used is the clear culture supernatant, filtered and concentrated (20 mg protein/mL) in 50 mM sodium acetate buffer, pH 5.0, with 0.05% w/v sodium azide added as biocide. The enzyme solution was stored at 4°C for months without loss of protein or activity. The specific activity of the cellulases was determined as 8.5 ± 0.9 U/mg. It was measured at 50°C using bacterial cellulose (BC; 1.0 mg/mL) as the substrate. Preparation of the BC is described below. β-Glucosidase (*Aspergillus niger*; Megazyme; Wicklow, Ireland) was added to the mixture at 2.0 mg/L. Incubation was done at 50°C in 1.0 mL total liquid volume (50 mM sodium acetate buffer, pH 5.0) in a 1.5 mL Eppendorf tube under shaking (500 rpm) using a Thermomixer comfort (Eppendorf AG, Hamburg, Germany). The glucose released after 1 h was quantitated enzymatically (D-Glucose HK Assay Kit, Megazyme).

2.2 Cellulosomes

The cellulosome is from *Clostridium thermocellum* ATTC 27405 and was obtained as described in earlier work.[2] The cleared culture supernatant was further purified by size exclusion chromatography. The typical composition of the fully formed *C. thermocellum* cellulosome was verified by SDS PAGE, confirming the presence of the main enzymatic subunits as well as the scaffold protein of ~197 kDa size. The cellulosome preparation was concentrated to ~0.15 mg/mL and stored in 30 mM MOPS buffer, pH 7.0, containing 100 mM NaCl and 10 mM $CaCl_2$. Enzyme solutions were stored at 4°C for months without loss of protein or activity. When performing reactions, the "cellulosome buffer" (30 mM sodium acetate buffer, pH 5.5, containing 100 mM NaCl, 10 mM $CaCl_2$, 10 mM cysteine and 2.0 mM EDTA) was always prepared fresh. Using the same conditions as described above, except for different buffer and temperature of 55°C, cellulosome had a specific activity of 3.1 ± 0.2 U/mg.

2.3 Disassembled cellulosomes

The ensemble of enzymatic subunits released from the scaffold protein is referred to as disassembled cellulosome.[2] This was prepared from the native cellulosome by a reported procedure, following the published protocol exactly.[2] Removal of the scaffold protein was verified by SDS PAGE. The preparation (~0.1 mg/mL) was stored similar to cellulosome. It was stable for at least 4-5 months. The specific activity of the disassembled cellulosome was

measured as for the native cellulosome, using cellulosome buffer. It was determined as 1.39 ± 0.2 U/mg.

2.4 Bacterial cellulose

Standard (unlabeled) BC. The BC is from *Acetobacter xylinum* (DSMZ-46602) and was obtained as described in earlier work.[2] The organism is grown on the medium 360. YPM from DSMZ. Note that the main carbon source is here mannitol. The BC film after 14 days of growth was harvested, clarified (1 M NaOH overnight at 4°C) and subsequently neutralized to pH 7.0 by repeated washing with Milli-Q water. It was then disintegrated using a hand blender for 1-2 min at room temperature. The resulting BC fibers were washed by several cycles of centrifugation (4500 g for 10 min at 4°C, Eppendorf, table-top centrifuge 5804 R) and resuspended in the reaction buffer for cellulase or cellulosome. The suspension of BC fibers was sonicated with a Sonic Dismembrator instrument (Fisher Scientific, GmbH) until it appeared homogeneous. The sonicated BC preparation was stored wet at 4°C and used as a substrate without any further modification.

^{13}C-labeled BC. This is *A. xylinum* BC produced in the 360. YPM medium from above, except that mannitol was replaced by uniformly ^{13}C-labeled glucose (Eurisotop, Saint-Aubin, France) in the same concentration of 25 g/L. The primary culture was obtained by inoculating a single colony of *A. xylinum* into 10 mL of the modified growth medium in 50 mL Corning tubes and incubating at 30°C under static condition for 7 days. The main culture (200 mL medium in 1 L unbaffled Erlenmeyer flasks) was inoculated (1%, by volume) and incubated statically at 30°C. *A. xylinum* grows slowly under the conditions of glucose carbon source, consistent with literature,[3] and the ^{13}C-labeled BC films were harvested only after 28 days of cultivation.[3] The ^{13}C-BC films were processed identically as the unlabeled BC up to the stage of disintegration with hand blender. The suspension of ^{13}C-BC fibers was lyophilized and stored at -20°C until use. The lyophilization step was necessary for preparation of concentrated stocks of ^{13}C-BC (≥ 7 g/L) in suspension. For use in enzymatic reactions, the lyophilized ^{13}C-BC was weighed into the reaction buffer and allowed to rehydrate at 4°C overnight. The resulting ^{13}C-BC suspension was homogenized using an instrument Palssonic Eco Ultraschallreiniger bath sonicator (Allpax, Papenburg, Germany) for 15 min at room temperature.

2.5 Enzymatic conversion of BC

Reactions were performed at different mass ratios of enzyme and substrate (E/S ratio, mg/g). The BC concentration was constant at 1.0 g/L unless mentioned otherwise. Variation of E/S was due to variable enzyme concentration in the range 0.25 – 8.0 (cellulases, cellulosome) and 1.0 – 13 (disassembled cellulosome). Incubations were made in a total liquid volume of 1.0 mL

(cellulase or cellulosome buffer) in 1.5 mL Eppendorf tubes using a Thermomixer comfort. Agitation was at 500 rpm, and the temperature was 50°C (cellulases) or 55°C (cellulosomes). β-Glucosidase (2.0 mg/L) was added to all reactions to hydrolyze soluble cellooligosaccharide products to glucose.

Homogeneous suspension of BC substrate was preincubated to reach reaction temperature. Reactions were started with the enzyme solution. Samples (80 μL) were taken at certain times and the reaction was stopped with NaOH to a final concentration of 100 mM. The samples were centrifuged (16900 g, 10 min at 4 °C, centrifuge 5804 R). The glucose in the supernatant was measured enzymatically. The BC conversion (%) at different times was calculated from the anhydroglucose released. The initial rate was calculated from the linear part of the glucose time course. Under the conditions used, the glucose release was reasonably linear up to 1 h. Measurements after 1 h were used.

2.6 Partitioning of enzymes between unlabeled and ^{13}C-labeled BC

Experimental set-up. The experiment was designed in which the ^{13}C-BC is made to compete with unlabeled BC for reaction with enzymes. Incubations were performed in 1.5 mL total liquid volume (cellulase or cellulosome buffer) using 2.0 mL Eppendorf tubes. β-Glucosidase was added at 2.0 mg/L. In the first step, enzymes (~1.0 mg/L) were incubated with 1.3 mL of 1.2 g/L unlabeled BC for 30 min. The E/S ratio was chosen low (~0.83) so that effectively all enzyme was adsorbed to BC under the conditions used. The amount of enzyme loaded (~18 nmol/g) is well below the maximum productive binding capacity of the major cellulase Cel7A (~122 nmol/g) on BC.[4] Then, 200 μL of 7.5 g/L ^{13}C-BC suspension was added. The addition left the original substrate concentration almost unchanged (1.1 g/L) and established equal concentrations of ^{13}C-BC and unlabeled BC (with enzyme adsorbed on it). Homogeneous samples were withdrawn at certain times, heated to ~100°C to stop the reaction (10 min), and centrifuged (16900 g for 10 min at 4°C, centrifuge 5804 R). The supernatant was analyzed for total glucose using the enzymatic test kit. The samples were additionally analyzed by LC/Q-TOF MS to determine the isotope ratio of unlabeled (^{12}C) glucose and uniformly ^{13}C-labeled glucose. The absolute concentrations of ^{12}C- and ^{13}C-glucose were determined from the ^{12}C/^{13}C isotopologue ratio and the total glucose concentration released. To check for cross-contamination of ^{12}C- and ^{13}C-glucose isotopes in ^{13}C-labeled and unlabeled BC, the ^{13}C-BC and the normal BC were hydrolyzed by cellulases and the isotope composition of the product was determined by LC/Q-TOF MS. The results validated that the unlabeled BC was comprised of ^{12}C-glucose, and the ^{13}C-BC of ^{13}C-glucose, each in an isotopic purity exceeding 99.5%.

$^{12}C/^{13}C$ isotope ratio measured by LC/Q-TOF MS. Isotope ratio data were acquired on an Agilent (Santa Clara, CA, USA) 6545 LC/Q-TOF MS with a dual jet electrospray ionization source system coupled to an Agilent 1290 Infinity II ultra-high performance liquid chromatography unit. Separation was achieved with an Agilent InfinityLab Poroshell 120 HILIC-Z column (2.1 × 100 mm, 2.7 µm) at 35°C. The mobile phase was 3% ammonium hydroxide in water (0.40 mL/min), and acetonitrile was used for isocratic elution at 80% (by volume). Autosampler injection volume was set to 0.5 µL and samples were cooled at 6°C. The analytical run time was 4 min.

Data analysis was conducted using the Agilent Personal Compound Database (PCDL) software. A custom PCDL was created for glucose, incorporating metabolite retention times. This custom database was subsequently used for batch isotopologue extraction in Profinder. The results from Profinder were exported for visualization in Microsoft Excel.

Batch isotopologue extraction in Profinder utilized retention time, accurate mass, and chromatographic peak shape to minimize false positives. All isotopologues of glucose were manually reviewed to ensure accuracy. Corrected abundance or percent enrichment, including natural isotope correction, was used to evaluate reproducibility. To elucidate the detailed labeling pattern, individual isotopologue enrichment plots were generated. These plots revealed that the most abundant labeling occurred at carbon number m+0 for unlabeled glucose and m+6 for labeled ^{13}C glucose.

2.7 Kinetic model of enzyme partitioning between unlabeled and ^{13}C-labeled cellulose

The model is based on Langmuir-type adsorption on cellulose and is derived to describe the distribution of enzyme (see Fig. 2) on unlabeled ([BC]) and ^{13}C-labeled BC ([BCL]), as well as in solution. The enzyme distribution is calculated based on the ratio of glucose release rates (Eq. S1 and Eq. S2 for the release of unlabeled and labeled glucose, respectively), which were measured in the presence of β-glucosidase, and expressed in physical dimension of mmol/(L min). Individual k_{act} values (expressed as mmol/(mg min)) were determined from the total glucose release rate in individual experiments, assuming that all enzymes are adsorbed. Note that k_{act} is considered the mass-based apparent specific activity of the respective cellulase system. No distinction is made between the respective subclasses of cellulases present in both systems, which have different substrate specificities, activities, and molecular weights. This simplification is possible and even necessary, as the model represents the distribution of the overall catalytic performance, rather than individual enzyme activities. Furthermore, the assumption that all enzyme is adsorbed is reasonable given the low enzyme-to-substrate ratio

used, approximately 0.02 µmol/g, assuming an average molecular weight of 60 kDa, similar to the archetypal Cel7A from *T. reesei* (see refs.[4,5] for the binding capacity of typical cellulosic substrates).

At the start of the reaction, the total enzyme is adsorbed onto the unlabeled substrate, implying that $[E_{BCL}] \approx [E_s] \approx 0$.

$$\frac{d[G]}{dt} = k_{\mathrm{act}}[\mathrm{E_{BC}}] \qquad \text{Eq. S1}$$

$$\frac{d[GL]}{dt} = k_{\mathrm{act}}[\mathrm{E_{BCL}}] \qquad \text{Eq. S2}$$

The enzyme [E] expressed in mg/L is distributed as follows: adsorbed to unlabeled cellulose (Eq. S3), further divided into portions that are reversibly absorbed (E_{BC}) and irreversibly adsorbed (E_I); adsorbed on labeled cellulose (E_{BCL}) (Eq. S4); and in solution (E_S) (Eq. S5). The inclusion of adsorbed activity unable to desorb (E_I) was necessary to describe activity time courses that reached a steady state with respect to the activity distribution, however, without reaching an equal distribution of activity on labeled and unlabeled cellulose.

$$\frac{\mathrm{d[E_{BC}]}}{\mathrm{dt}} = k_{\mathrm{on}}[\mathrm{E_S}][\mathrm{BC}] - k_{\mathrm{off}}([\mathrm{E_{BC}}] - [\mathrm{E_I}]) \qquad \text{Eq. S3}$$

$$\frac{\mathrm{d[E_{BCL}]}}{\mathrm{dt}} = k_{\mathrm{on}}[\mathrm{E_S}][\mathrm{BCL}] - k_{\mathrm{off}}[\mathrm{E_{BCL}}] \qquad \text{Eq. S4}$$

$$\frac{\mathrm{d[E_S]}}{\mathrm{dt}} = -k_{\mathrm{on}}[\mathrm{E_S}][\mathrm{BC}] - k_{\mathrm{on}}[\mathrm{E_S}][\mathrm{BCL}] + k_{\mathrm{off}}([\mathrm{E_{BC}}] - [\mathrm{E_I}]) + k_{\mathrm{off}}[\mathrm{E_{BCL}}] \qquad \text{Eq. S5}$$

As the glucose release rate was constant, within the limit of error of ± 10%, upon the addition of labeled BC, it can be excluded that enzymes have become inactivated during the incubation in the time of the experiment.

Note that the concentrations of BC and BCL do not change appreciably (conversion: ≤ 10%) in the observed time span, hence $\frac{\mathrm{[BC]}}{\mathrm{dt}} \approx \frac{\mathrm{[BCL]}}{\mathrm{dt}} \approx 0$. Due to the constancy of [BC] and [BCL], the second-order adsorption rate constant, dependent on substrate concentration, is simplified to

k^*_{on} (Eq. S6) which is a pseudo first-order rate constant. Eq. S3, S4 and S5 thus reduce to Eq. S7, S8 and S9, respectively.

$$\mathrm{k^*_{on} = k_{on}[BC(L)]} \qquad \text{Eq. S6}$$

$$\frac{d[E_{BC}]}{dt} = k^*_{on}[E_S] - k_{off}([E_{BC}] - [E_I]) \qquad \text{Eq. S7}$$

$$\frac{d[E_{BCL}]}{dt} = k^*_{on}[E_S] - k_{off}[E_{BCL}] \qquad \text{Eq. S8}$$

$$\frac{d[E_S]}{dt} = -2k^*_{on}[E_S] + k_{off}([E_{BC}] - [E_I]) + k_{off}[E_{BCL}] \qquad \text{Eq. S9}$$

Rate constants of adsorption (k^*_{on}) and desorption (k_{off}) have the dimension of reciprocal time and are determined in min^{-1}. The adsorption-desorption rate constants are assumed to be independent of ^{13}C-labeling of BC. Glucose release may be affected by ^{13}C isotope slightly (≤ 5%) but not in an extent that would be relevant for the experiments here performed.

2.8 Data fitting

Modeling and fitting were performed in MATLAB R2023b. Data fitting was done using MATLAB's lsqnonlin (nonlinear least-square method with trust-region-reflective algorithm) utility utility available in the MATLAB optimization toolbox. Data sets containing the activity distribution for the respective enzyme preparations were analyzed and the fit parameters were k_{off} and E_I. The k_{on} was fixed at 10^4 min^{-1} to allow for immediate re-adsorption of released enzyme (i.e., $E_I \approx 0$). This step was necessary as the model assumes constant activity on the surface, or as it is known from literature, k_{on} is typically one up to multiple orders of magnitude faster than k_{off}.[6–8]

Starting values for the fit parameters were randomly selected, and iterative fitting was performed, using MATLAB’s default step size tolerance (10^{-6}) and a maximum of 400 iterations. Based on the obtained parameter values and residuals, a 10% confidence interval was calculated for cellulases and cellulosomes. Due to the rapid equilibrium establishment and fully reversible binding observed for the disassembled cellulosome, confidence interval estimation was deemed unnecessary in this case.

2.9 Specific hydrolysis rate of adsorbed enzyme

Specific activity of the adsorbed enzyme is referred to as R_{ads} with the physical dimension of $\mu mol_{glucose}/(mg_{enzyme}\ min)$. R_{ads} is the ratio of the glucose release rate $R_{glucose}$ in µmol/(L min) and the adsorbed protein concentration P_{ads} in mg/L. $R_{glucose}$ and P_{ads} are temporal variables of the BC hydrolysis, as described in earlier.[9] R_{ads} also changes with time or relatedly, conversion. $R_{glucose}$ is determined by interpolation between two times of sampling as Δ[glucose]/Δt. P_{ads} is the adsorbed protein concentration at interpolation start.

We carefully examined various BC concentrations (1 – 2.2 g/L) and enzyme loadings (17 – 25 mg/L) to identify which are suitable to accurately measure the temporal evolution glucose and protein in solution during BC hydrolysis. For cellulases, 1.9 g/L BC and 17 mg/L enzyme (E/S 8.95) were used. For the cellulosome, 1.65 g/L BC and 20 mg/L enzyme (E/S 12.12) were used. Reactions were supplemented with β-glucosidase (2.0 mg/L). Reactions were conducted in a total liquid volume of 1.4 mL in 2.0 mL Eppendorf tubes. Agitation was at 600 rpm using a Thermomixer comfort. At certain times, 150 µL of homogeneous sample was withdrawn and centrifuged (12400 g for 5 min at 4°C). Fifty µL of the clear supernatant were heat-treated (100°C, 10 min) and assayed enzymatically for total glucose. The remaining 100 µL were used to measure the soluble protein, using Roti-Nanoquant assay calibrated with BSA. The background from soluble β-glucosidase was considered in the calculation of P_{ads}. Background from the cellulosome buffer, presumably effect of the Cys reducing agent, was also corrected. Data was collected from at least three independent reactions and the SD was indicated in the error bars.

2.10 Enzymatic reactivity of partially hydrolyzed BC

Partially degraded preparations of the BC were obtained to assess change in substrate reactivity towards enzymatic hydrolysis (cellulases, cellulosome) as the conversion by enzymes progresses. To minimize the processing required after partial degradation of the BC (e.g., to remove the enzyme used), the enzymatic hydrolysis was performed at low E/S ratio (≤ 1.75) for longer periods of time (≥ 12 h). Time courses of enzymatic conversion (Fig. S5) were used for guidance.

Incubations were made in 50 mL Corning tubes that contained 30 mL total volume of 1.0 g/L BC. A shaking water bath (GFL-1083, Gesellschaft für Labortechnik, Burgwedel, Germany) was used for agitation (150 rpm) and temperature control. The BC suspension was preincubated for 15 min and enzyme (cellulase: 0.5 mg/L, 50°C; cellulosome: 1.75 mg/L, 55°C) was added. β-Glucosidase was added (2.0 mg/L) additionally. Incubation was continued at the temperature for up to 19 h. The reaction mixture was centrifuged (4500 g for 10 min at 4°C, centrifuge 5804

R) and the supernatant was used to measure glucose for determination of degree of conversion. The residual BC was washed thoroughly (up to five times) with 30 mL of buffer and then stored at 4°C until used further within 24 h. Additionally, after cellulosome treatment, the residual BC was subjected to a thermochemical treatment to remove the bound cellulosomes.[2] The BC was washed (three times) with 50 mM sodium acetate buffer, pH 5.0, containing 25 mM EDTA and heated at 70°C, for 20 min. The mixture was centrifuged (4500 g for 10 min at 25°C) and the pellet was resuspended in EDTA-containing buffer. This disassembly step was repeated three times after which the BC was washed again (three times) with cellulase reaction buffer. Hydrolysis experiments were done as described above to determine R_{ads}.

2.11 Atomic force microscopy (AFM) – nanomechanical characterization of cellulose deconstruction

AFM measurements were done in a buffer droplet (250 µL) using a Dimension Fast Scan Bio™ atomic force microscope (Bruker, Berlin, Germany). The measurement parameters were as follows, adjusted to the requirement of the system under investigation. Cellulosomes were monitored at 35°C using FastScan D probes (Bruker AFM Probes, Camarillo, CA, USA) with nominal frequency, spring constant and tip radius of 110 kHz, 0.25 N/m and 5 nm, respectively. Cellulases were observed at 35°C using FastScan D probes or at room temperature using PEAKFORCE-HIRS-SSB probes (Bruker). Nominal frequency, spring constant and tip radius were 100 kHz, 0.12 N/m and 1 nm, respectively. Temperature was adjusted to balance enzyme loading and degradation rate, without altering the fundamental activity of the enzyme system.

The preparation of BC substrate on a highly ordered pyrolytic graphite (HOPG) block (grade I) from SPI supplies (West Chester, PA, USA) was done as reported in earlier work.[10] In brief, a freshly cleaved HOPG surface (about 1 cm^2) was incubated with 300 µL of diluted BC suspension (<0.01 g/L) for 15 min. The crystal was rinsed with deionized water, and any remaining droplets were eliminated by briefly spraying carbon dioxide. The crystal was mounted onto the liquid stage of the AFM and coated with 250 µL buffer to avoid contamination and drying of the adsorbed cellulose fibers.

After recording reference images, the degradation experiment was started by injecting 10 µL of enzyme solution (100 µg/mL) into the droplet. Data evaluation was done using Gwyddion (Version 2.55)[11] and MATLAB 2023b.

Before each measurement, probe deflection sensitivity was measured on HOPG and spring constant were calculated via the thermal tuning method. The effective tip radius was estimated based on the nominal tip radius and the tip opening angle provided by the manufacturer. The scanning size was either 1 × 1 µm or 1 × 0.5 µm and digital resolution was set to either 2 × 2

or 4 × 4 pixel per nm². For imaging, the peak force set point was set to either 2.5 nN for cellulosomes. Cellulases were observed using peak forces ranging from 0.3 to 2.0 nN, depending on the experiment and tip used. The applied peak force regime was chosen to ensure reliable imaging of the bacterial cellulose surface while minimizing artifacts. Direct comparison with previous AFM studies on soft matter in liquid is limited, as those often used amplitude setpoints instead of fixed peak force.[12]

A Poisson's ratio of 0.3 was assumed for cellulose,[13,14] which is consistent with literature values. The tip modulus was assumed infinite. The cantilever was oscillated at 2 kHz, and the scan rate was adjusted to maintain a tip velocity below 1 µm/s. Force-distance curves were automatically analyzed in real time using Brukers PeakForce QNM mode. During the measurement, the operator monitored the force–distance curves to ensure proper tip–sample interaction (Fig. S6).

The dynamic region of the retract curve (Fig. S6, purple dashed line) was fitted using the Derjaguin–Muller–Toporov (DMT) model to obtain the reduced modulus. Given the Poisson's ratio, this value can be converted to the sample Young's modulus (E), which quantifies material stiffness, i.e. the resistance to deformation under stress. The DMT model is appropriate for our measurements because the AFM tip can be approximated as a rigid sphere on an elastic plane, the observed deformations are predominantly elastic, and the bacterial cellulose films are sufficiently thick and laterally unconstrained to minimize boundary effects. PeakForce QNM can provide quantitative modulus values over 700 kPa to 70 GPa, provided the probe is calibrated and the DMT assumptions are satisfied. If the tip-sample geometry deviates strongly from a sphere-on-plane contact, if non-elastic deformation mechanisms dominate, or if the sample is laterally or vertically confined, the modulus map remains qualitatively useful, as demonstrated in the technical note by the manufacturer (Bruker).[15]

2.12 BC fibers and their elementary unit of nanostructure

Observations were done on the fundamental unit of the BC nanostructure which is the microfibril. This microfibril consists of 18-36 individual cellulose chains, based on previous studies.[7,16,17] In the BC substrate used, several of these microfibrils are arranged in fiber bundles that form the basic three-dimensional architecture of this cellulose. The fiber bundles consist of quasi-repetitive units comprising more or less straight sections interrupted by regularly occurring twists (1-2 per µm).[2]

These quasi-elementary (or repeating) units were selected for imaging and further analysis. Additional criteria were that the selected fibers were relatively intact, straight, and free of significant nanoscale defects such as breaks and kinks. The typical length of the defined

elementary unit is approximately 500 nm, and the corresponding fiber width typically ranges between 10 and 30 nm, indicating that areas of about 5000 to 20000 nm^2 (depending on pixel size) were examined.

2.13 AFM data structure and workflow

AFM data are organized in a three-dimensional matrix. The scanned surface is represented by the lateral x–y plane, where each pixel corresponds to a defined area with nanometer-scale dimensions. In the z-dimension, several layers are stored, each containing a distinct property of the same x/y position. In this study, two primary channels were used: (a) topography, reporting the absolute pixel height derived from the Z-piezo position, and (b) the Young's modulus (*E*), calculated from the tip–sample interaction by the software as described above.

To enable quantitative evaluation, raw AFM images were further processed in a structured workflow (**Fig. S7**). First, the image was prepared for subsequent analyses by background correction and removal of undefined particles (Step 1, **Fig. S7**, see also section 2.14.). Next, the region of interest was segmented into fiber and background. This mask was refined by removing small, isolated "islands" not belonging to the main fiber structure (Step 2, **Fig. S7**, see also section 2.15). The measured modulus values were then normalized to the arithmetic mean of the HOPG background, yielding normalized stiffness values (E^*), which served as an internal reference (Step 3, **Fig. S7**, see also section 2.16). Finally, space-averaged modulus values (E^*_{av}) were obtained by averaging all E^* values within the masked fiber region (Step 4, **Fig. S7**, see also section 2.16) and analyzed. In parallel, the fiber height was tracked over time using the corresponding topography channel (see section 2.18 onwards), as described in earlier studies,[2,10] enabling later correlation of E^*_{av} with enzymatic degradation.

2.14 AFM data acquisition and processing for analysis of *E* modulus and derived parameters

Acquisition of AFM images of elementary fiber units was performed as described in Sections 2.11 and 2.12. Depending on the fiber dimensions, image size, and geometry, up to two elementary units could be captured in a single measurement. Multiple units were recorded per experiment; however, only fibers that were completely degraded (residual volume < 20% of initial volume) were included in the analysis. The typical time resolution was ~0.3 frames per minute, with ≥12 frames acquired per elementary unit (corresponding to ~35 min).

Raw AFM images meeting the criteria were first corrected using the Gwyddion software package.[11] Corrections included adjustment of the height channel to remove tilt and enable automated fiber detection. Typically, a first-order linear correction was applied. Alternatively, the difference in medians of measured lines was used, followed by a planar fit if residual tilt

remained. Any unspecified particles on the surface (e.g., non-degradable structures or contaminants) were manually identified, marked, and removed (Fig. S8 A, B). Importantly, the *E* modulus dataset was not altered during these corrections except for removal of such particles. The modified height and raw *E* modulus images were then exported as 2D matrices and imported into an in-house MATLAB routine.

2.15 Fiber masking and *E* extraction from Height and Modulus Matrices

The matrices generated above were loaded into an in-house MATLAB routine to mask fibers and separate them from the HOPG background. Each pixel in the height matrix was classified as either a "fiber pixel" or a "background pixel" based on its height. A binary mask was generated using the median height of the image as a threshold: pixels below the median were assigned to the background, while the remaining pixels were assigned to the fiber (Fig. S8C). Small, isolated features ("islands") below a typical size threshold of 150–300 pixels were excluded from the analysis.

Mask accuracy was verified by comparing the masked and original height images. If the mask did not appropriately represent the fiber, the threshold was iteratively adjusted. Manual adjustment was particularly necessary in later stages of enzymatic conversion, when changes in fiber height and morphology reduced the reliability of automated detection.

Once the fibers were accurately masked, the corresponding *E* values were extracted into a vector, losing their direct x/y positional information, and processed further.

2.16 Calculation of the space-averaged *E* modulus (E^*_{av})

To account for variations in AFM tip properties (e.g., changes in tip radius or laser drift) and to enable comparison across measurements, all raw *E* modulus values were normalized. To account for variations in AFM tip properties (e.g., slight changes in tip radius or laser alignment) and to enable comparison across multiple measurements, raw *E* modulus values were normalized in a structured procedure. First, the arithmetic mean of the HOPG background (unaffected by enzymatic activity) was calculated for each image. The mean background modulus was then kept constant across all frames, using the value from the first reference image as the internal reference.

Next, the arithmetic mean of the fiber region was computed for each frame. The fiber mean was then shifted proportionally to the constant background mean, so that all changes were expressed as a ratio relative to the stable HOPG reference (Fig. S9). The resulting normalized stiffness value, referred to as E^*, allows for direct comparison of stiffness distributions both across time-lapse frames and between different experiments (Fig. S9 and S10). Subsequently, to assess substrate stiffness, the space-averaged E^* modulus—referred to as E^*_{av}—was calculated. This

was obtained by computing the arithmetic mean of all E^* values within a defined fiber region (i.e., the "fiber pixels").

2.17 Statistical evaluation and quality control of elementary fiber units

Elementary fiber units (see section 2.12), typically containing ~2500–5000 data points (~10,000–20,000 nm²), were selected for statistical evaluation. These units were assessed for internal homogeneity (Fig. S11) based on the mean and shape of their E^* distributions (Fig. S12, Table S2). The distribution curves consistently exhibited a Gaussian profile with a shoulder on the left side, likely corresponding to disordered or loosely bound surface material. Units with irregular distributions were excluded from further analysis.

Larger fiber units (≥3000 pixels) were split into two halves to evaluate internal consistency. In representative examples, both halves showed nearly identical E^*_{av} values and distribution shapes (16.31 ± 1.10 vs. 15.56 ± 1.38 MPa and 19.59 ± 1.76 MPa vs. 19.09 ± 2.07 MPa; Fig. S11A and B, respectively), confirming the homogeneity of the selected regions. The initial distribution of such a unit before enzyme addition (at -6 min) is shown in Fig. S11A.

For fibers that became heavily degraded due to enzymatic activity (conversion ≥75%), automatic masking frequently failed, necessitating manual segmentation in Gwyddion. This approach also helped reduce the "bottom effect"[18] by restricting the mask to the innermost region of the cellulose crystal.

To enable comparison across different AFM tips and enzyme systems, all E^*_{av} values were normalized on a linear scale: the highest observed value in each experiment was set to 100%, the lowest to 0%, and intermediate values were linearly interpolated. This approach allowed for relative, qualitative comparisons across varied conditions.

Occasional drift or cropping during AFM imaging caused minor shifts in the analyzed areas (≤10%). Given the structural similarity of the preselected fiber units and their demonstrated internal consistency, these deviations were considered negligible (Fig. S11 and S12).

2.18 AFM data processing and correction of AFM time-lapse images for pixel-based calculations

The AFM images depicted in the time-lapse videos and utilized for pixel-based calculations underwent preprocessing using an automated MATLAB routine, as detailed in our earlier publications.[2,10]

The routine was devised based on the typical successive correction steps commonly performed in established software such as Gwyddion or Nanoscope (Bruker). It comprised the following main steps: object masking, background leveling, scaling, drift correction, and analysis. All topography data (height channel) underwent processing in this manner.

Identification and segmentation of objects (cellulose) and background (HOPG) were executed by detecting edges and surfaces using user-defined gradient and median parameters. These parameters were set based on the first image of a sequence, with manual parameter adjustment until over 90% of the pixels were accurately assigned to either object or background. Once suitable parameters were established, they remained unchanged throughout the sequence, resulting in individual masks for each frame within a sequence, calculated with consistent settings. Associating each image frame with its respective mask allowed for consistent image corrections.

The first correction step addressed tilts in the images by computing a plane that best fit the 3D data points defined by all pixels designated as background in the masking step. A plane was subtracted from the entire image (background + object pixels) to correct the tilt.

Next mismatched baselines of rows in the fast scan direction were corrected by fitting a first-degree polynomial to the background pixels using the least square method. Fitted lines were sequentially subtracted from all pixels in individual rows, excluding rows consisting of > 80% object pixels to avoid introducing calculation artifacts.

Consistent false-color scaling was achieved by setting the lowest value for every image to zero, followed by defining a user-selected maximum value to which all images were scaled.

Corrected and scaled datasets were exported in their original pixel size in portable network graphics (.png, 24-bit depth) for movie generation and as 2D matrices containing the corrected height information for further analysis.

2.19 AFM movie construction and .png image generation

Prior to movie construction the exported .png images were drift-corrected using a previously developed routine[2,10] where a user-selected reference image was compared to all other images in the sequence, determining the optimal shift in the *x*- and *y*-directions for each image to best match the reference image. The cross-correlation function between each image and the reference images was utilized to ascertain the best match. Movies were constructed from the drift corrected images using ImageJ 1.56g (rsbweb.nih.gov).

2.20 AFM based degradation analysis

The quantitative analysis of global volumetric degradation was performed in MATLAB using drift-corrected .png images containing height information. First, the RGB images were converted to 8-bit versions and cropped to the area that remained consistently within the scanned region. This step is necessary because drift correction can cause fibers at the edge of the scanned area to not always lie uniformly within it. This means that, in contrast to the dataset

used to determine the E^*_{av}, the dataset used to determine the volume loss is slightly cropped (typically < 10%) for volume calculation.

The modified .png dataset is then processed, and the described routine for marking objects (fibers) is applied to identify the cellulose fibers. For each image under the mask, the sum of all pixel entries was calculated, with the sum of the first image defined as 100%. The temporal development of this percentage represents the global degradation behavior of the enzymes under investigation (i.e., a reduction in the sum of all pixels indicates a loss of material). This loss of material was used to calculate the relative activity (Rel. Act.) according to Eq. S10 and expressed as % per min.

$$Rel.Act. = \frac{Pixelsum_{t1} - Pixelsum_{t2}}{t_2 - t_1} \qquad Eq.S10$$

'Pixelsum' refers to the sum of all pixel entries under the mask at a given time (indicated by the subscript), which identifies cellulose fibers. To compare degradation rates from different experiments, the degradation rate (i.e., Rel. Act.) was normalized as follows: the highest degradation rate (% per min) was defined as 100% for both systems combined, with no degradation defined as 0%. Intermediate values were calculated assuming a linear slope. Note that this approach allows for qualitative comparison between the two systems but does not provide conclusions about absolute rates. Since the degradation rates and the E^*_{av} data were obtained from nearly the same dataset (see above), these data can be directly correlated.

3 Supporting Figures and Tables

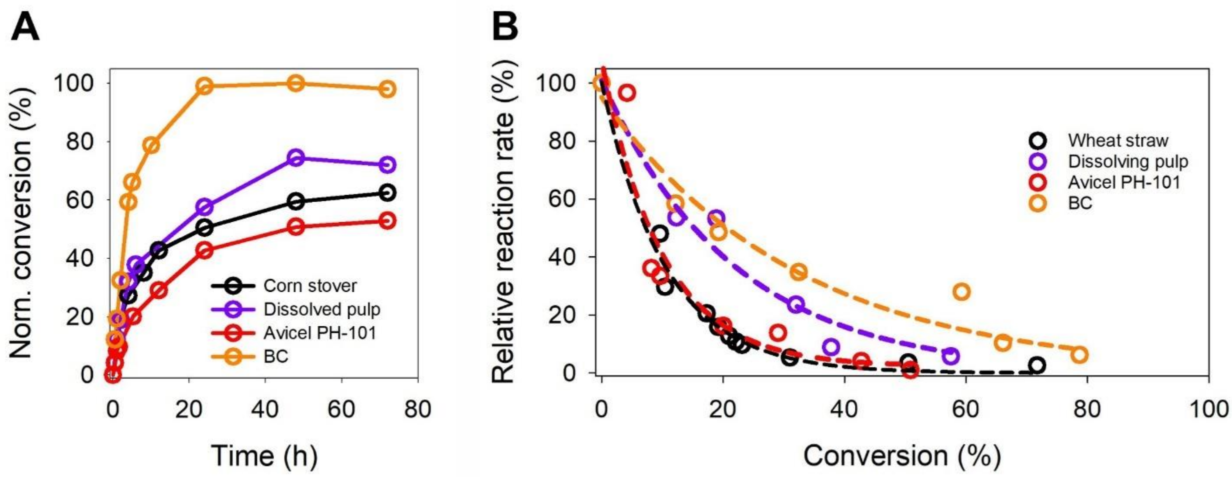

3.1 Fig. S1.

Enzymatic degradation of different cellulosic substrates, displaying the slowdown effect. (A) Time courses of conversion of various (lignocellulosic) substrates, revealing the slowdown to affect the maximum degree of degradation. (B) Depiction of the decrease in reaction rate with increasing conversion. Refer to Table S1 for sources and properties of the different cellulosic substrates used.

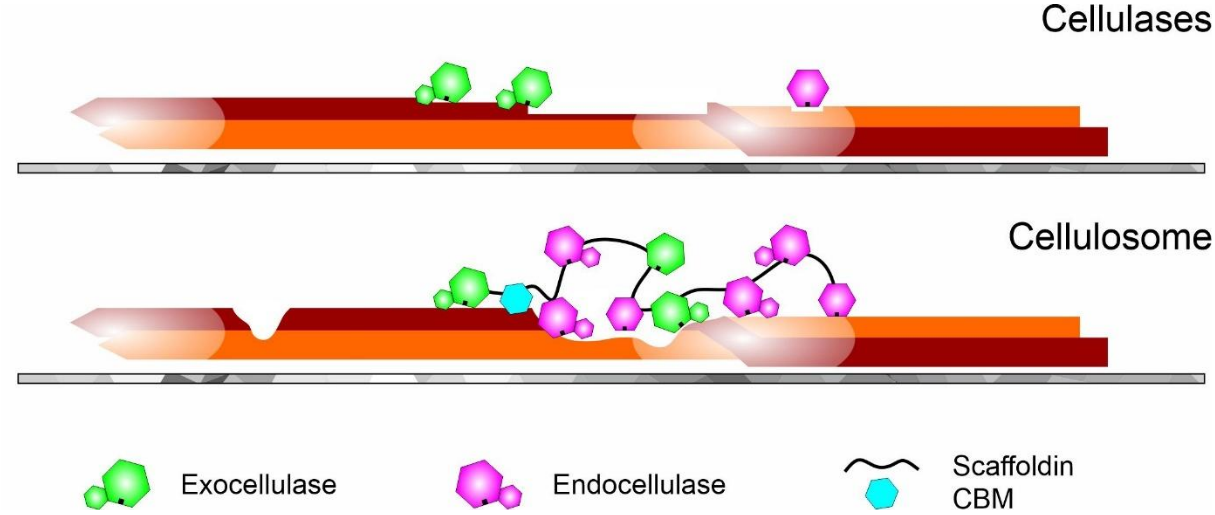

3.2 Fig. S2.

Schematic representation of cellulose organization and enzymatic degradation mechanisms. Two twisted (micro-)fibrils are shown within a fiber (bundle), alongside two major cellulase systems responsible for cellulose depolymerization. The upper panel illustrates dispersed individual cellulases,[7] including processively acting exocellulases that cleave cellulose chains from the ends, and endocellulases that introduce internal cuts to generate new chain ends. The lower panel depicts a cellulosome,[19] a multi-enzyme complex composed of coordinated cellulolytic subunits. Carbohydrate-binding modules (CBMs), associated with both systems, facilitate substrate binding.

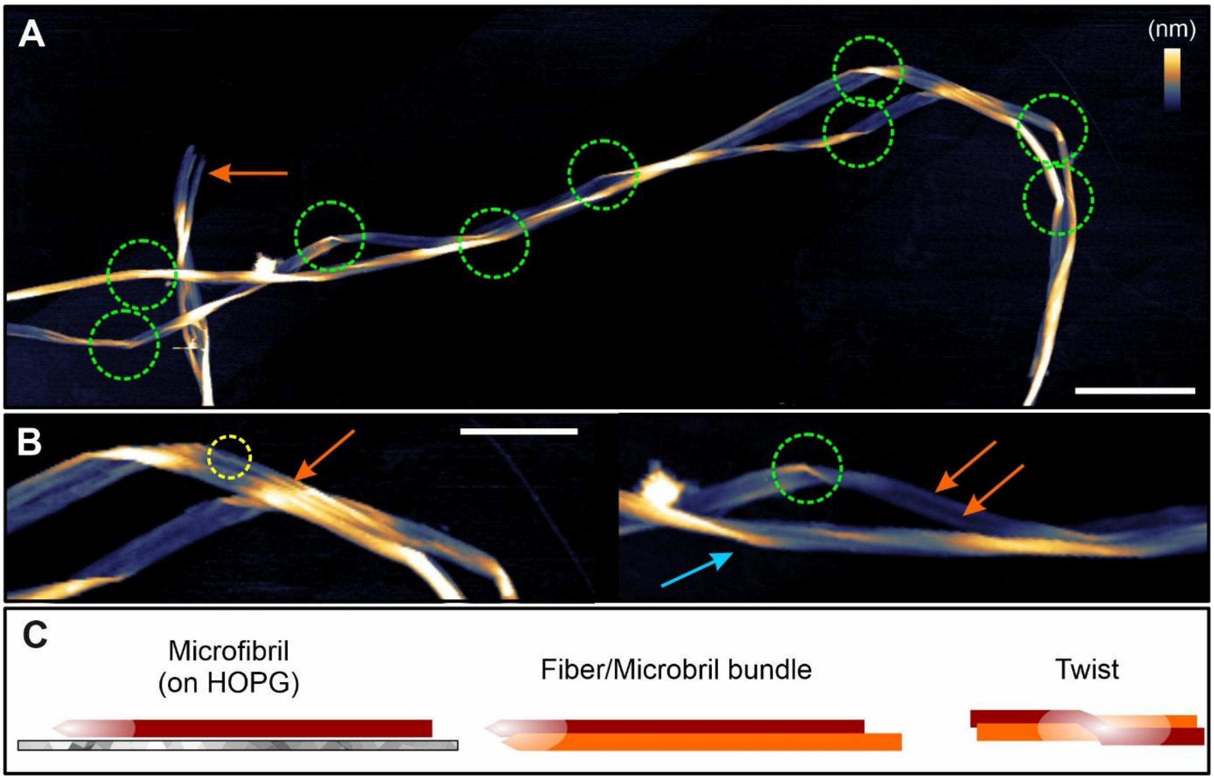

3.3 Fig. S3.

(A) Representative cellulose fiber bundle composed of multiple aligned fibrils. Straight bundle fragments occasionally display local twists, where one fibril crosses another within the same bundle (examples highlighted in green). Individual fibrils are typically exposed at bundle termini (orange arrow). (B) Magnified view of two linear fibril bundles composed of clearly discernible fibrils (orange arrows). Local irregularities occur at the level of individual fibrils (yellow circle). Twists appear at different length scales, ranging from short (green circle) to extended segments (cyan arrow). (C) Schematic representation of the terminology used throughout this work. Softer or less ordered regions—such as fibril ends or amorphous junctions—are shown in lighter colors.

Scale bars are 500 nm (A) and 250 nm (B). False-color scales are included for the AFM images in panel A. Height range is 25 nm (A), 30 nm (B, left) and 35 nm (B, right), respectively.

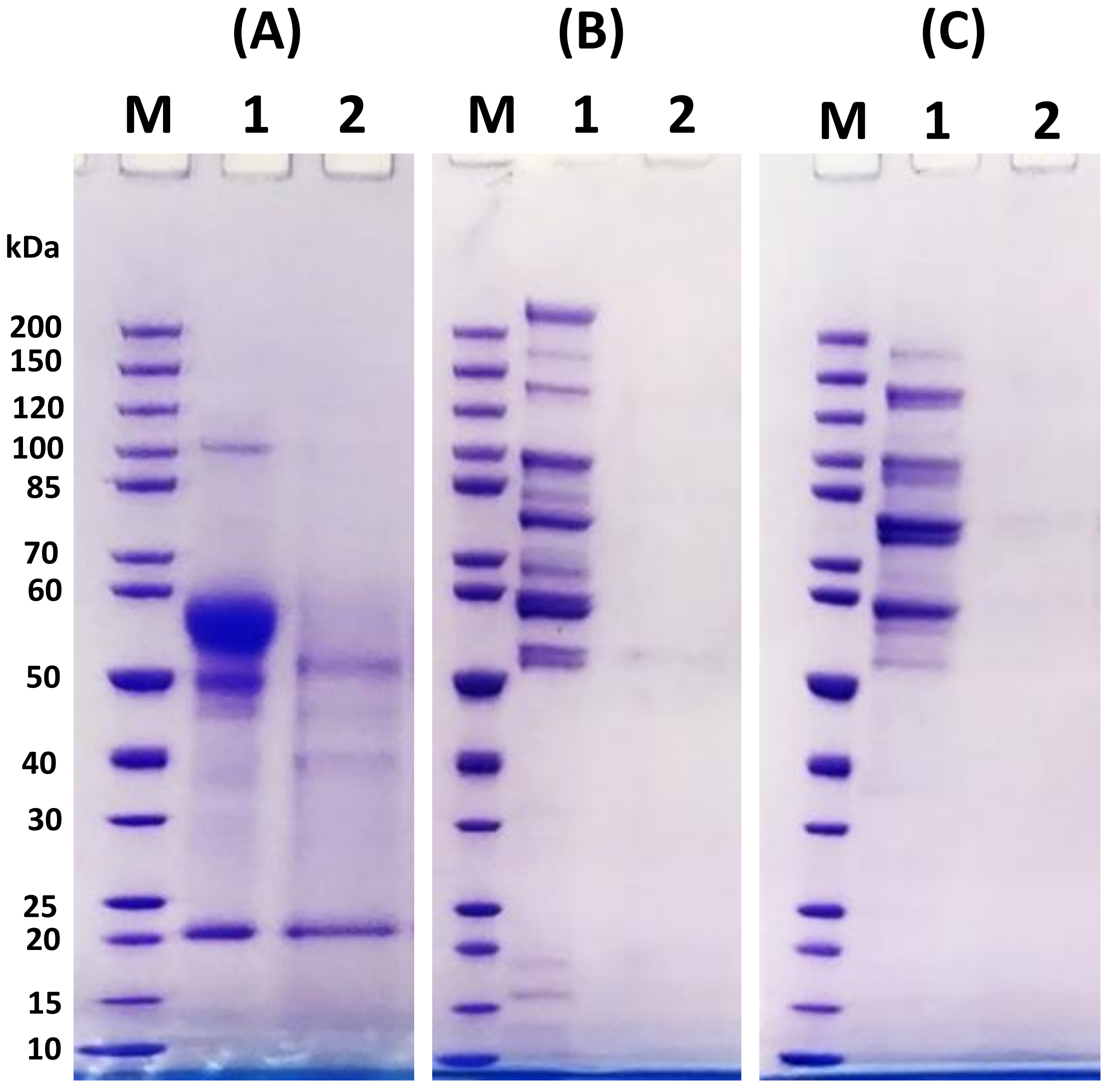

3.4 Fig. S4.

SDS PAGE analysis of non-adsorbed proteins in the supernatant after 1 h adsorption. Cellulases (A), cellulosome (B), and disassembled cellulosome (C). Lane 1: standard protein preparations used for BC adsorption; Lane 2: corresponding non-adsorbed enzyme fractions. Both lanes were loaded with ~5.5 µg protein/well. To promote adsorption and minimize hydrolysis, enzymes (20 µg/mL) were incubated with BC (1.24 mg/mL) at a reduced incubation temperature (22°C) under shaking (600 rpm). Analysis by ImageJ indicated that non-adsorbed enzyme fractions of cellulases at equivalent protein load to standard preparation can only be detected at < 5%. However, the cellulosome and disassembled cellulosome remained below detection.

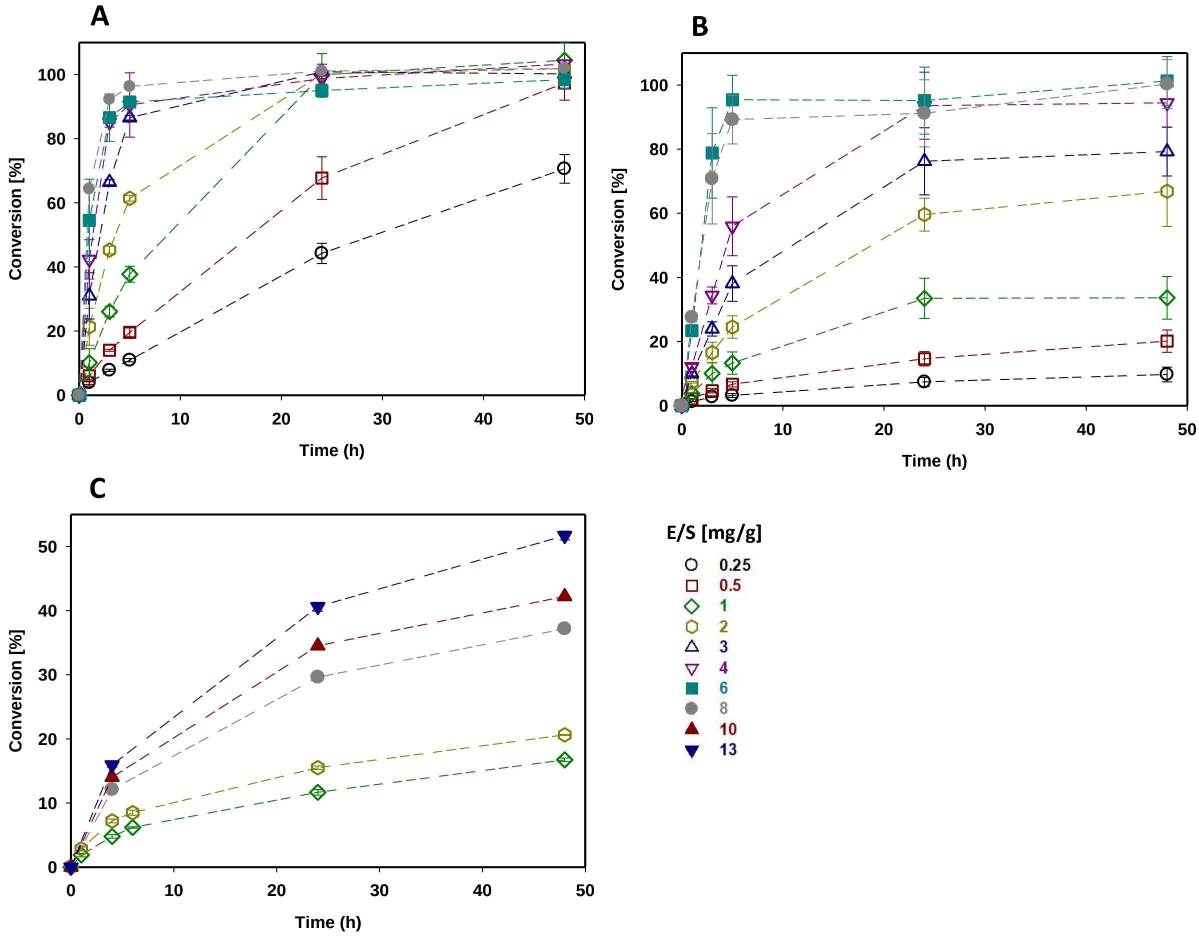

3.5 Fig. S5.

Cellulose conversion at varying loading of cellulases (A), cellulosome (B), and disassembled cellulosome (C). The BC concentration was 1.0 g/L. Symbols corresponding to different E/S loadings are shown in the inset. Reactions (n=3) were performed under optimum conditions of cellulases and cellulosome. See the section 2.5 for further details.

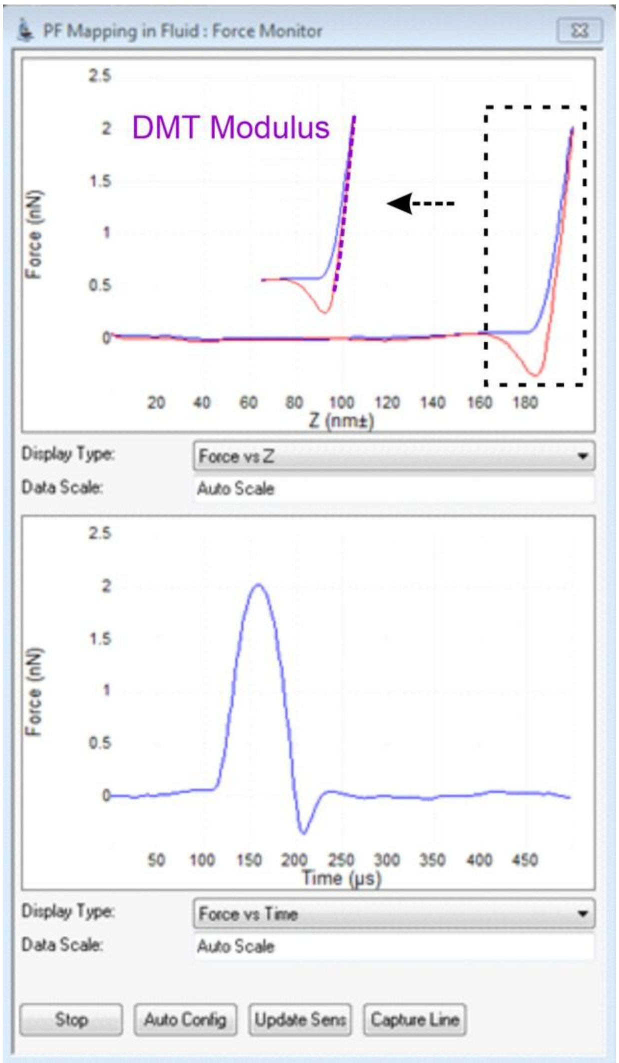

3.6 Fig. S6.

Force–distance and force–time profiles characteristic of PeakForce measurements on BC. Force–distance curve showing the interaction force (nN) as a function of the vertical position of the cantilever (Z). The blue curve represents the approach of the AFM tip toward the sample surface, while the red curve corresponds to the retraction (top panel). The inset highlights in purple the region used for calculating the DMT modulus. Force–time curve recorded during one PeakForce tapping cycle, illustrating the periodic nature of the tip–sample interaction (bottom panel).

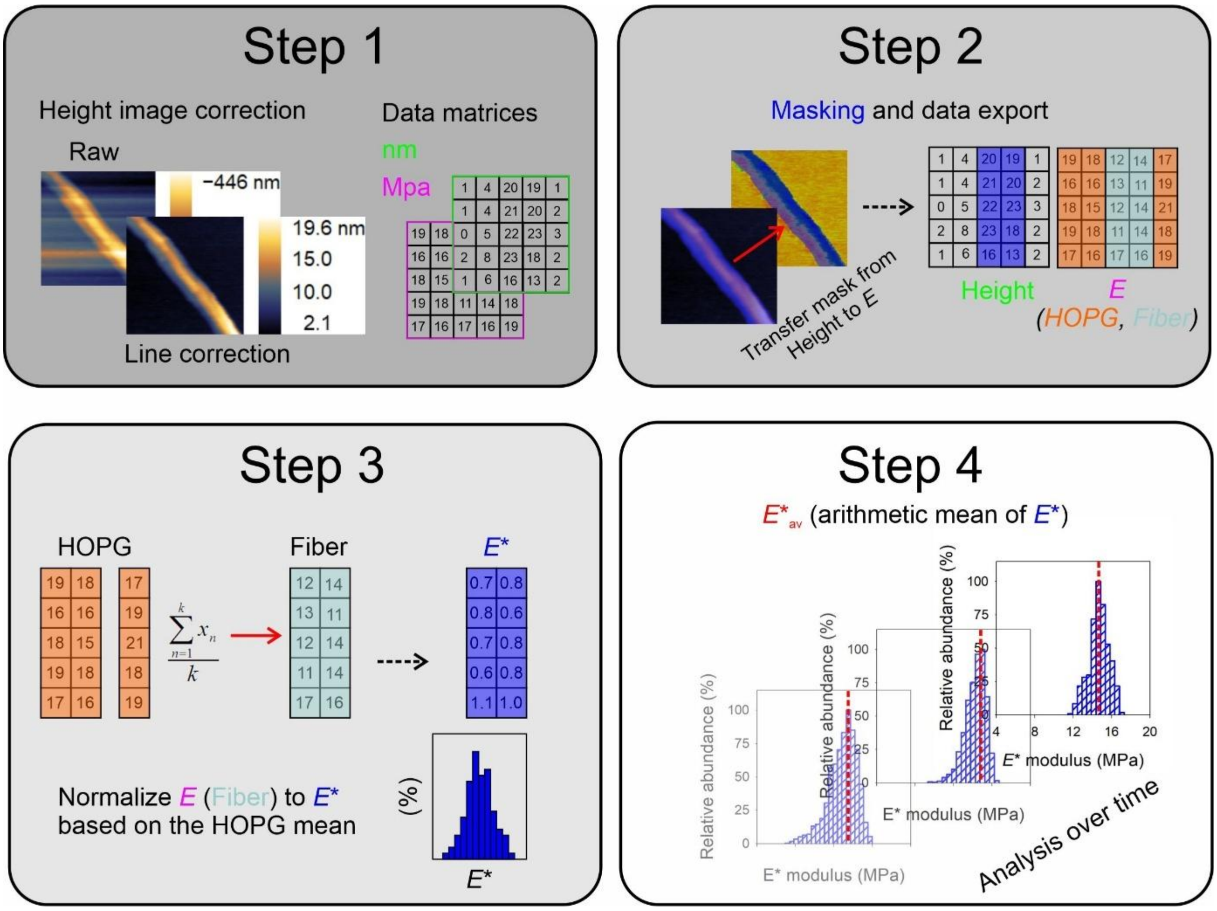

3.7 Fig. S7

Workflow for quantitative analysis of AFM nanomechanical maps. Step 1: background correction and removal of undefined particles. Step 2: segmentation into fiber and background with refinement of the mask. Step 3: normalization of modulus values to the arithmetic mean of the HOPG background. Step 4: calculation and analysis of space-averaged modulus values (E^*_{av}) from the masked fiber region.

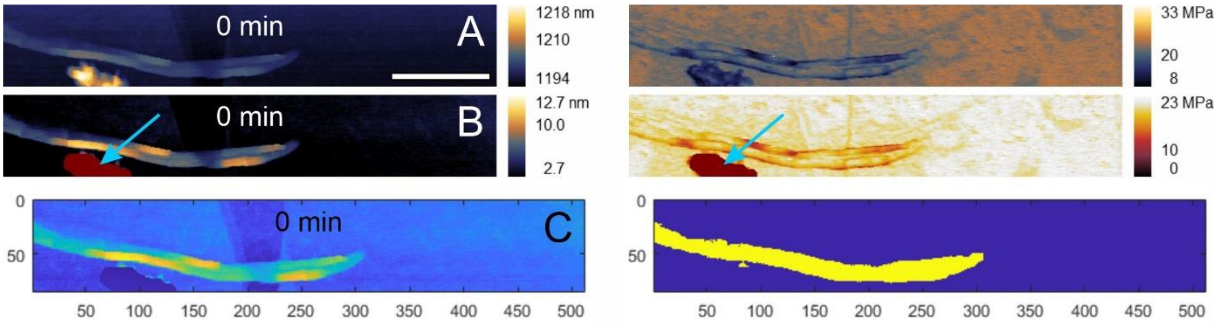

3.8 Fig. S8.

Data processing for *E** modulus analysis. (A) AFM raw data for height (left panels) and *E** modulus channel (right panels) at three different time points. (B) The same images as shown in panel A (0 min mark) after background correction (only applies to the height channel). Finally, loose undefined material was removed from the data set (red mask, indicated by arrows). (C) The AFM height channel after being processed by our in-house developed MATLAB routine (left panel) alongside the corresponding calculated mask (right panel). Note that the length is now given in Pixel (image dimensions 512 × 85 pixel).

Scale bar is 200 nm and false color scales are included for the AFM images. Note that the color scales in panel A were not adjusted (i.e., they remain as recorded during the experiment) to facilitate better comparison between the raw data and the corrected versions in panel B.

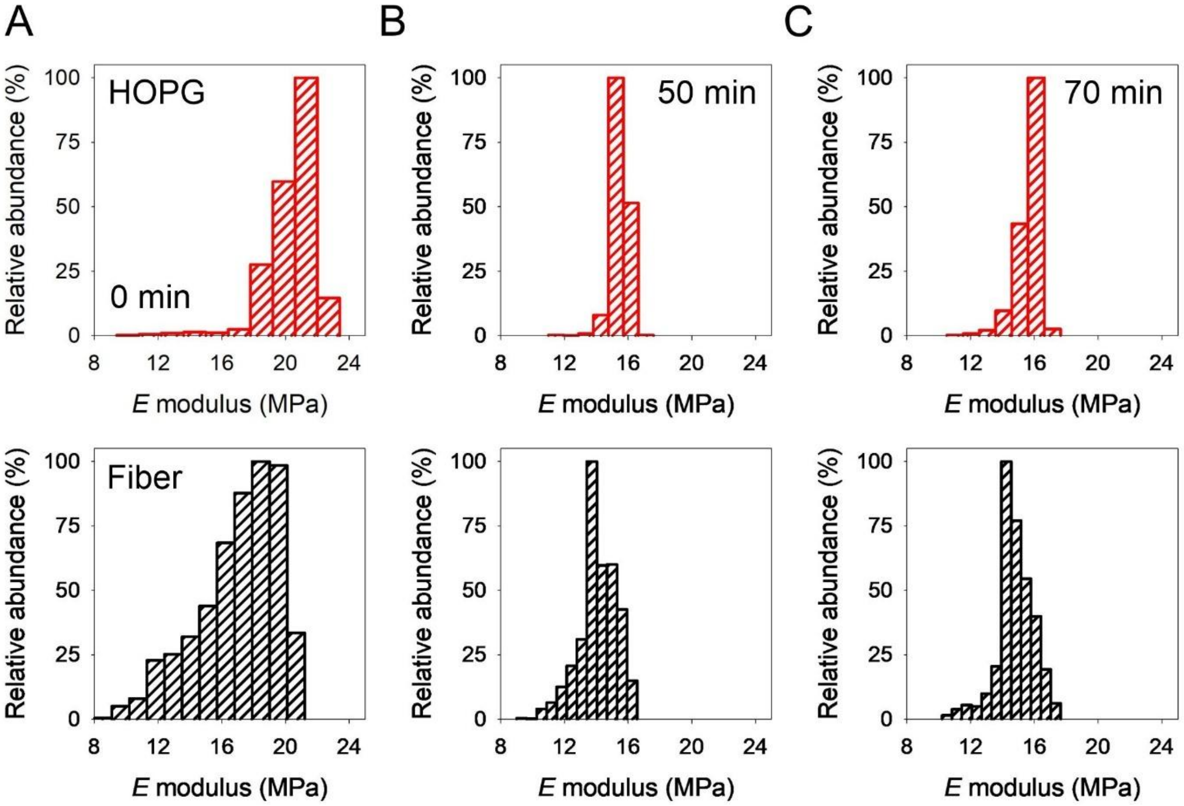

3.9 Fig. S9.

Extracted *E* modulus distributions from the analyzed fiber (masked in yellow) HOPG background shown in Fig. S8. The *E* modulus obtained for the background shifts over time (see top panels), likely due to enzyme/tip interference, a common occurrence for soft biomolecules.[20] Consequently, the *E* modulus values of the fiber (lower panels) for each image were considered as a percentage of the HOPG, which remains unaffected by enzymatic activity. The mean values of the HOPG were normalized to their initial value at time 0 min, and the *E** modulus of the fibers was calculated accordingly (see Fig. S7).

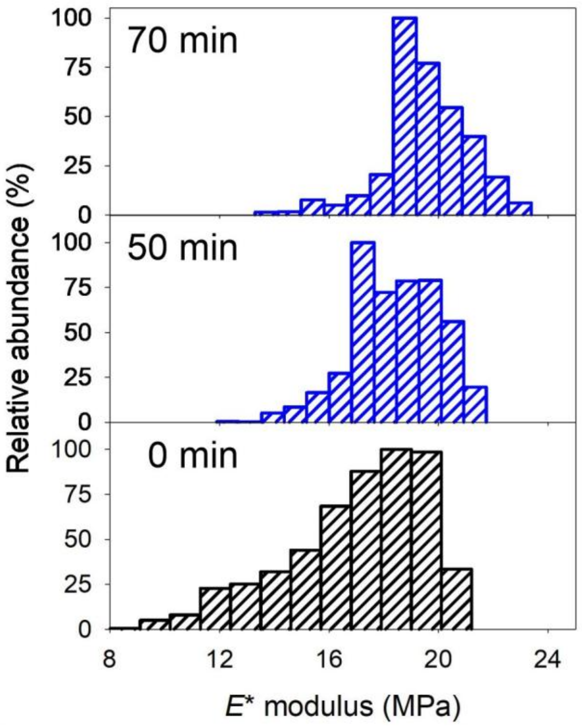

3.10 Fig. S10.

*E** modulus distributions of an analyzed fiber segment shown in Fig. S8A at multiple time points. The raw data can be seen in Fig. S9A-C (lower panels).

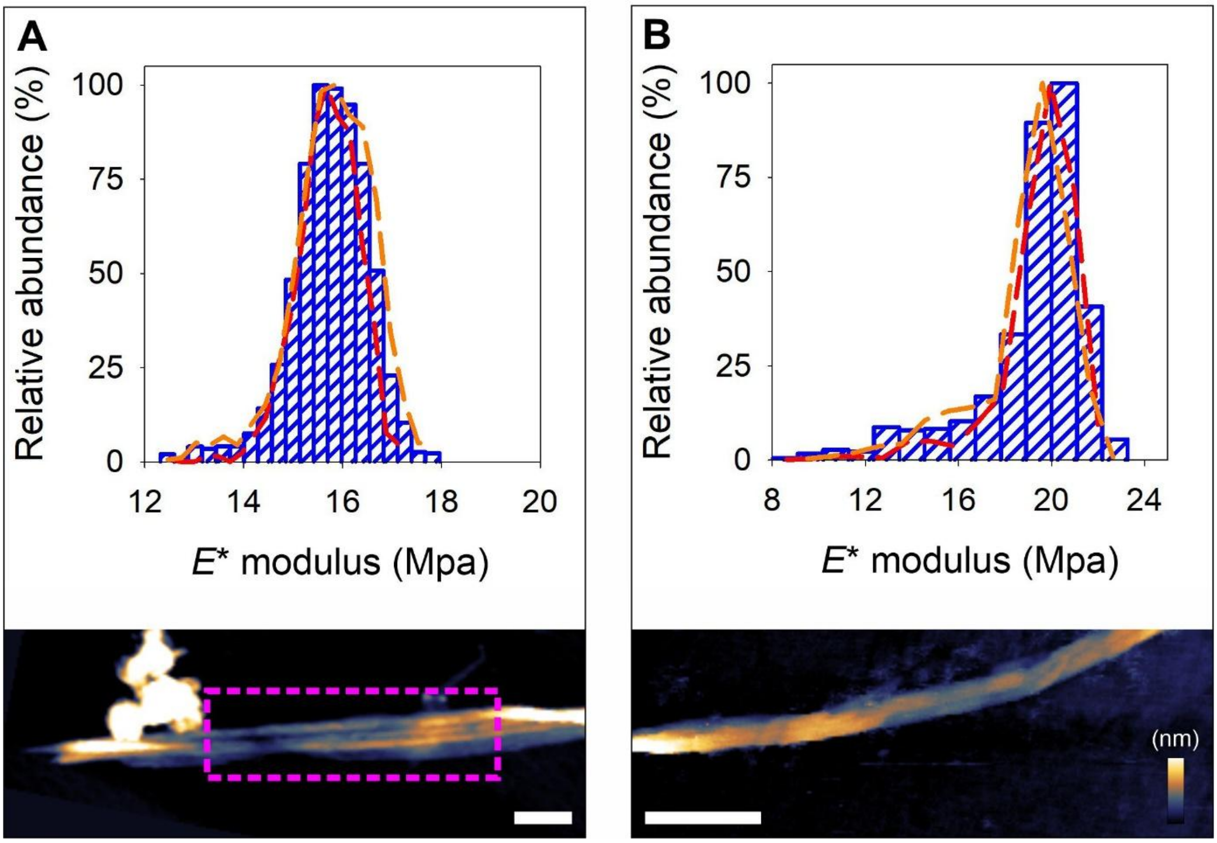

3.11 Fig. S11.

(A, B) The two subdistributions of *E** (dashed lines) exhibit trends similar to the overall distributions of the elementary units (bars), with no significant deviations. The elementary units used for analysis are shown at the bottom (pink rectangle in A). Scale bar: 100 nm. False color scale (shown in B) represents a height range of 15 nm in (A) and 10 nm in (B).

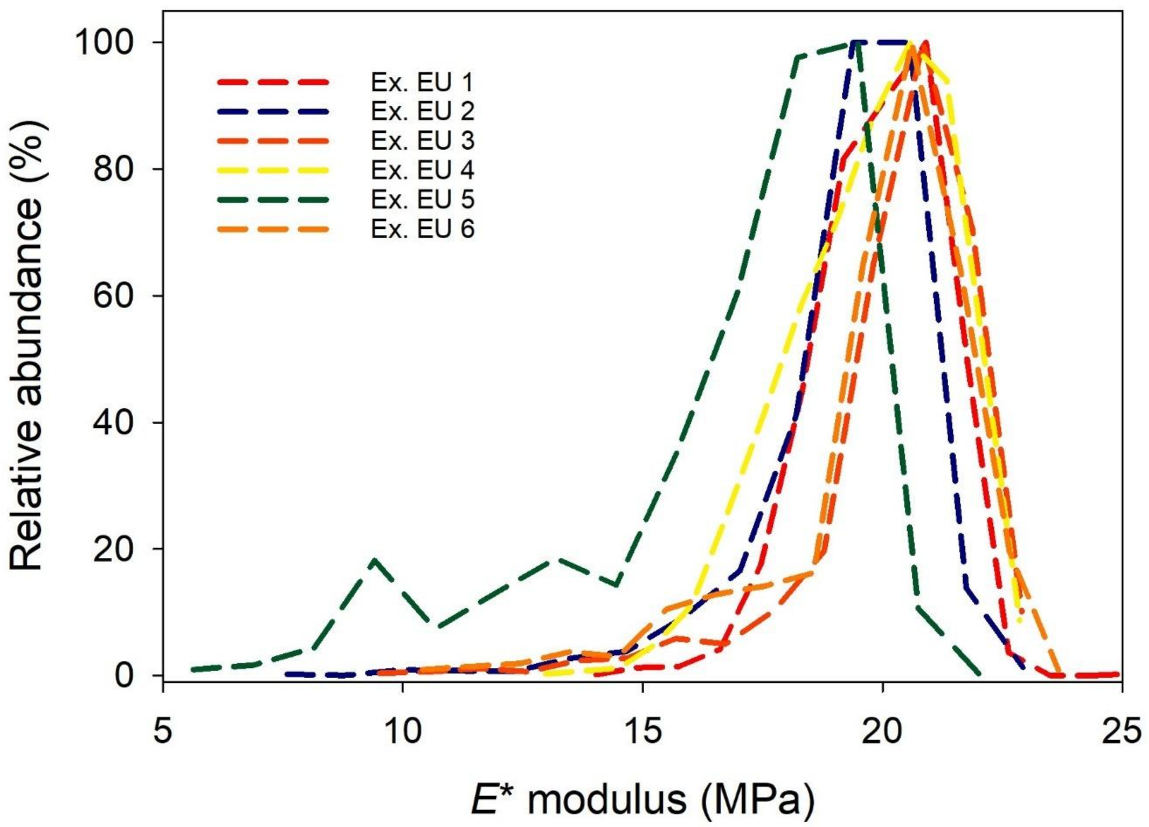

3.12 Fig. S12.

*E** modulus distributions of elementary units. All displayed units show consistent distribution patterns and fulfill the morphological criteria for analysis (section 2.14), including the absence of visible structural damage. All distributions were recorded using FastScan D cantilevers.

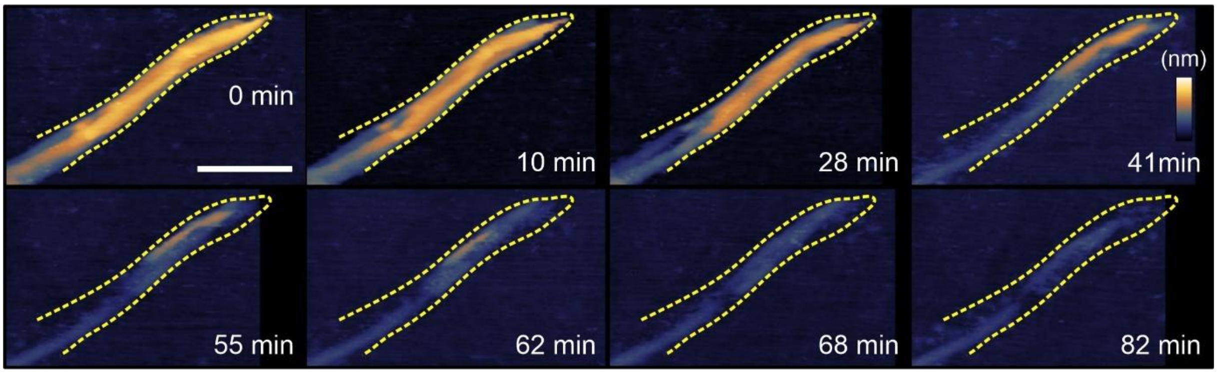

3.13 Fig. S13.

Time-lapse observation of elementary unit degradation by cellulases. The original width is highlighted in yellow. Additional images for this sequence can be seen in Fig. 4B.

Scale bar is 100 nm and the false color scale covers a height range of 20 nm.

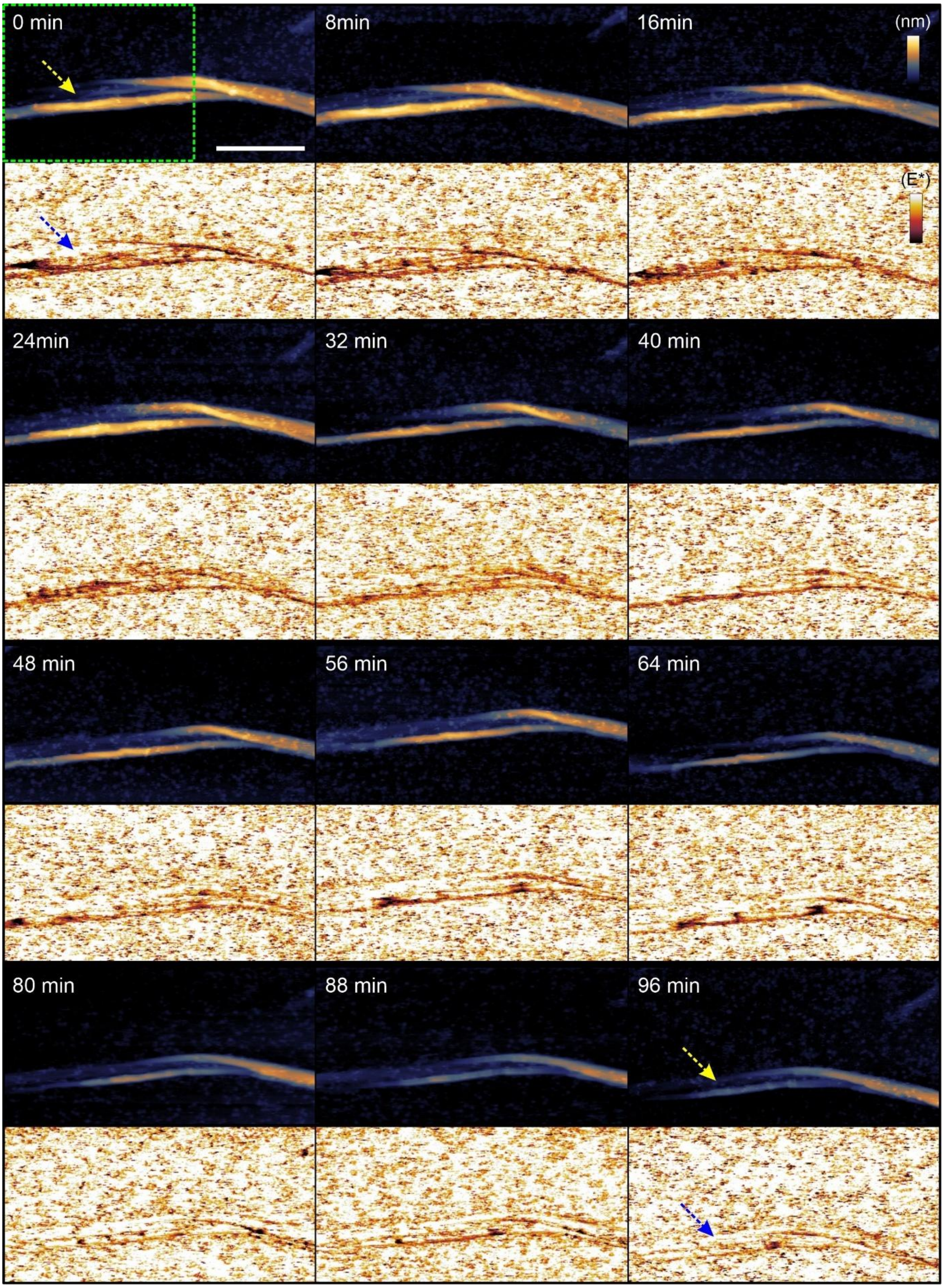

3.14 Fig. S14.

Time-lapse observation of BC fiber degradation by cellulases. Top panels show AFM height images; corresponding *E* modulus maps are shown below. A partial elementary fibril unit used

for quantitative analysis (see Fig. S19A) is highlighted by a green frame. The fibril marked by yellow and blue arrows illustrates progressive degradation accompanied by a relative increase in stiffness from the beginning to the end of the experiment.

Scale bar 100 nm and the false color scale covers a height range of 18 nm. E^* modulus maps are false-colored relative to the mean background stiffness, with the scale spanning from 50% below the mean upward. The mechanical response of the BC lies mostly within this normalized range.

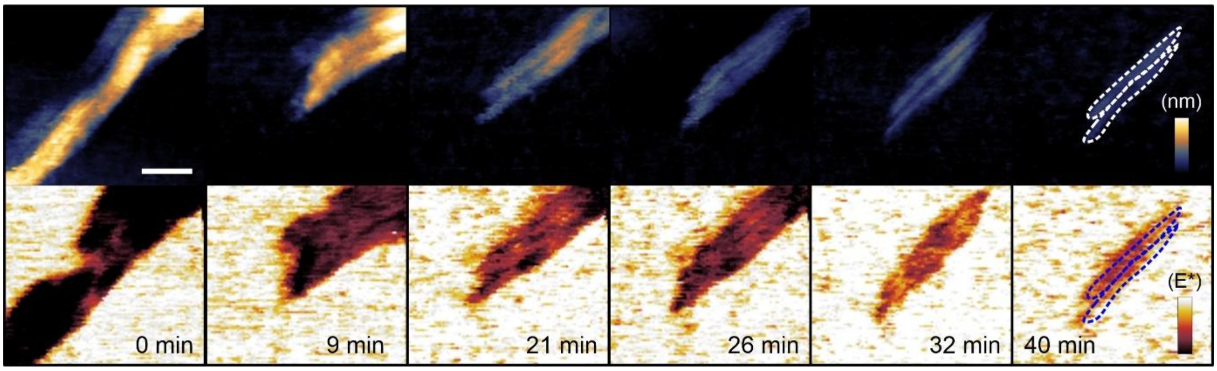

3.15 Fig. S15.

Time-lapse observation of BC fiber degradation by cellulases. Top panels show AFM height images; corresponding E modulus maps are shown below. Fibril thinning and flattening are observed over time, accompanied by a relative increase in stiffness. The remaining fiber material is outlined by a white/blue frame. Data shown were used for quantitative analysis shown in Fig. 5C. The full sequence is provided in Movie S2.

Scale bar 100 nm and the false color scale covers a height range of 18 nm. The false color scale for the $E*$ modulus was normalized to the mean stiffness of the background region, covering a range from 70% below to 10% above this mean. The mechanical response of the BC lies mostly within this normalized range.

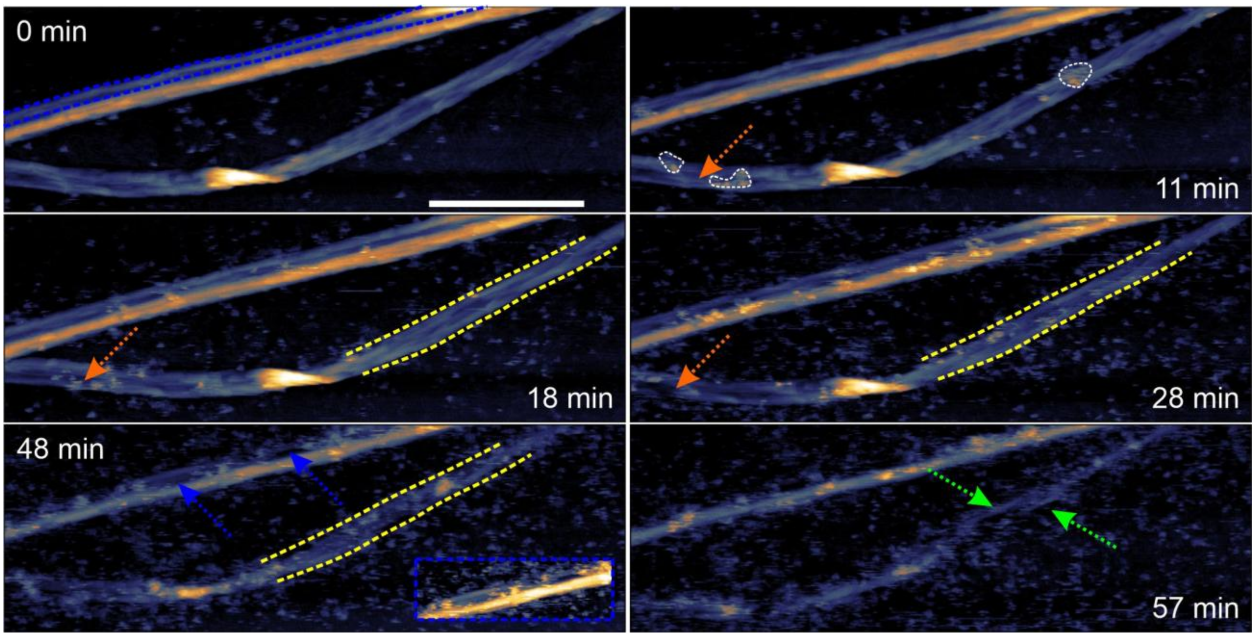

3.16 Fig. S16.

Time-lapse observation of BC fiber degradation by cellulosome. Exemplary cellulosomes on the BC fiber are circled in white at 11 min. Initial fragmentation without significant volumetric degradation is marked by orange arrows, and a cellulosome can also be observed at this location at the 18-min mark. A fibril, marked in blue at the 0-min mark, is fragmented after 48 min and only partially remains. The blue arrows point to two fragmented fibrils, which are more clearly visible with increased contrast in the inset (blue frame). The yellow frame highlights a fiber section where degradation occurs mainly through internal fragmentation. Green arrows point to thin isolated fibrils originating from this disrupted region, which likely represent the structural core of the cellulose fiber.

Scale bar is 250 nm and the false color scale covers a height range of 17 nm and 12 nm for the inlet (48 min mark).

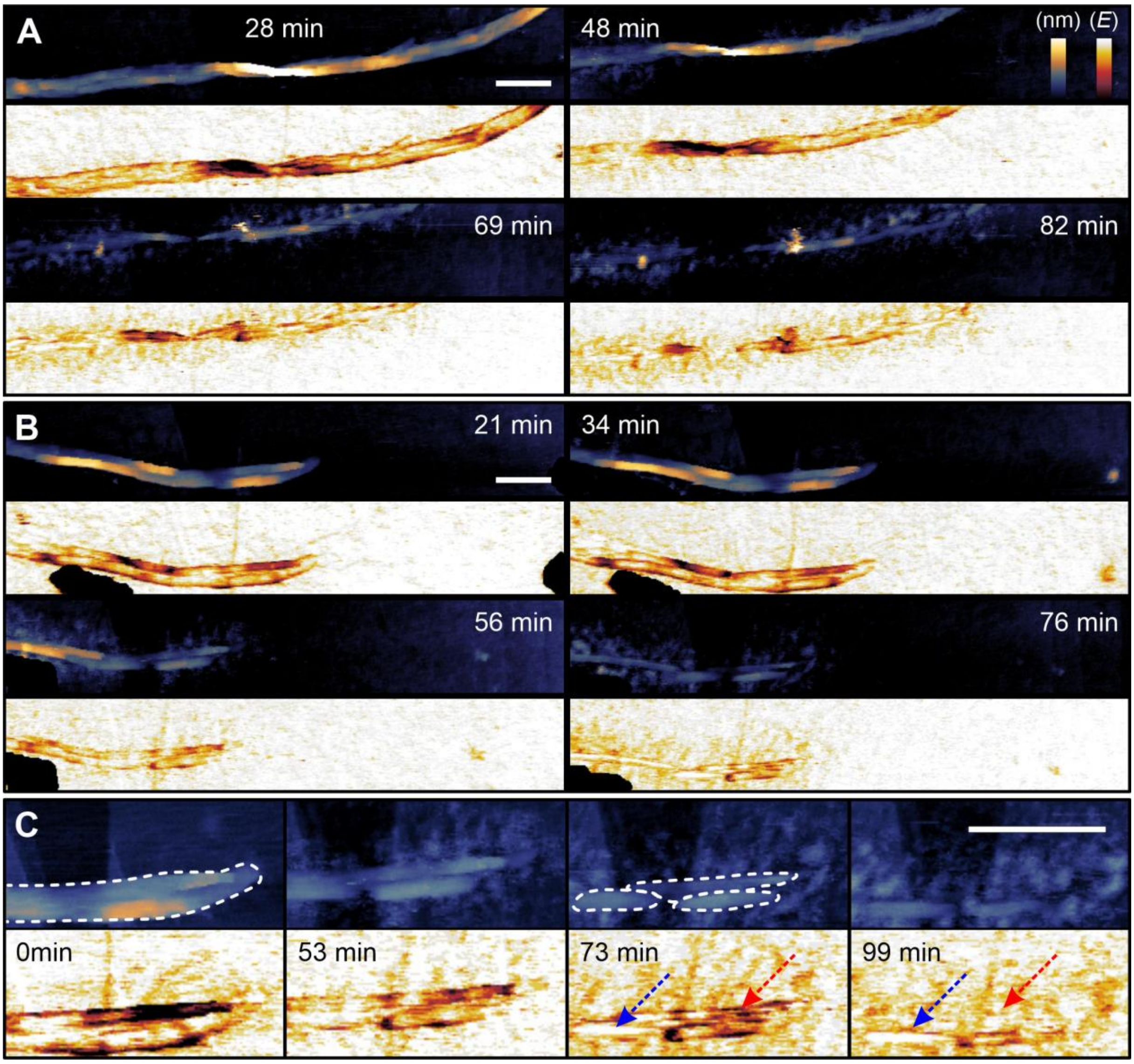

3.17 Fig. S17.

Time-lapse observation of BC fiber degradation by cellulosomes. Top panels show AFM height images; corresponding E^* modulus maps are shown below. Initial and final states of elementary units degraded ($\geq$ 80%) by the cellulosome (A, B). The snapshots shown here, along with intermediate time points (not shown), were used to generate the data presented in Fig. 5B, D and Fig. S19B, C, respectively. Panel C shows the late-stage fragmentation of fibrils into small crystalline fragments (indicated with arrows). Full sequences for panels A and B can be seen in Movie S4 (A) and S5 (B), respectively.

Scale bars are 100 nm (A, B) and 200 nm (C). The false color scale covers a height range of 10 nm (A, B) and 5 nm (C). E^* modulus maps are false-colored relative to the mean background stiffness, with the scale spanning from 50% below the mean upward.

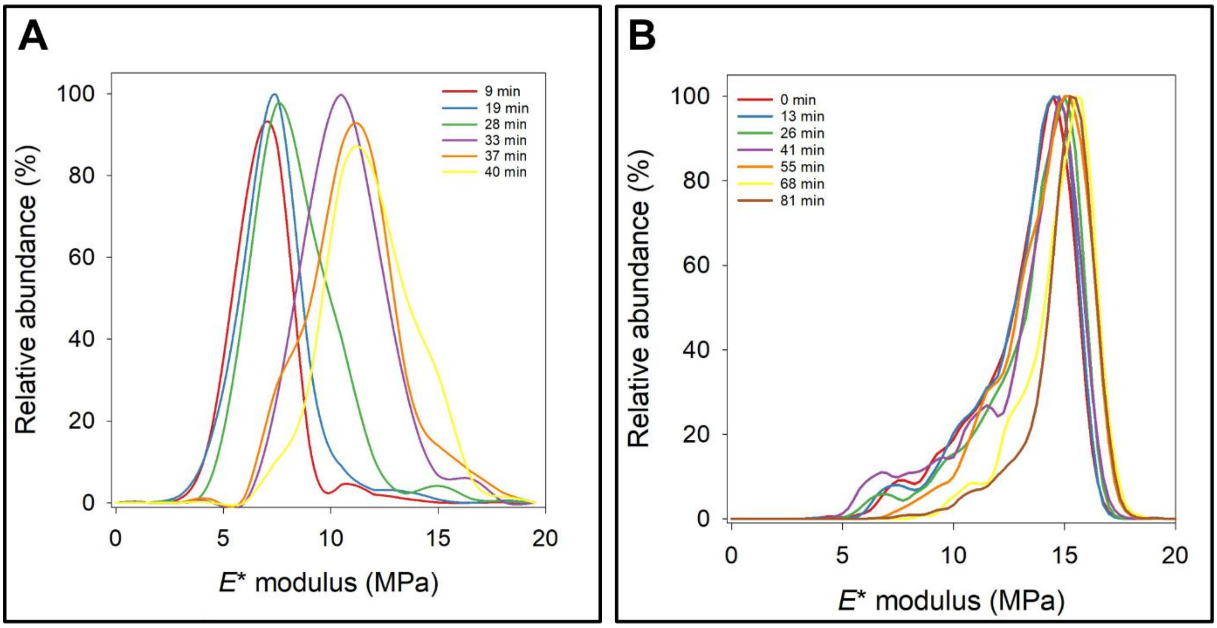

3.18 Fig. S18.

Exemplary evolving *E** modulus distributions during enzymatic degradation of (A) cellulase and (B) cellulosome. The corresponding E^*_{av} time courses are shown in Fig. 5C and 5D, respectively.

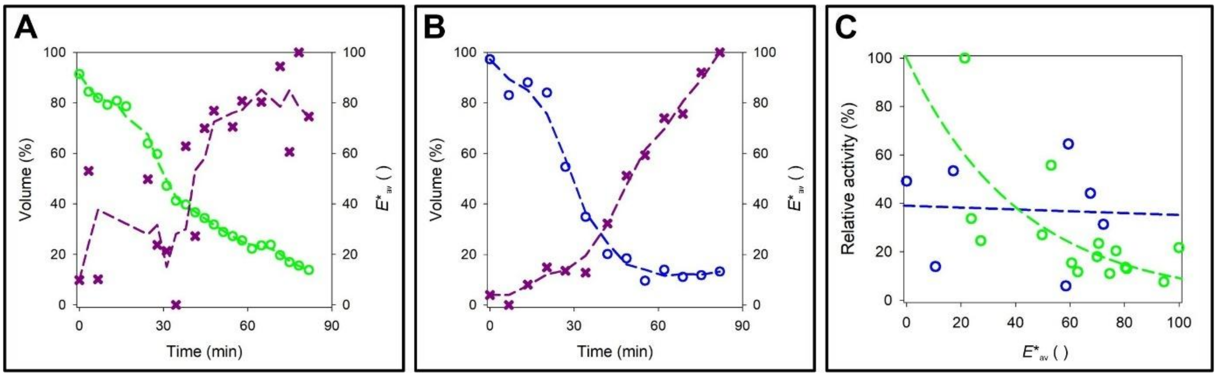

3.19 Fig. S19.

Temporal evolution of cellulose volume and E^*_{av} (both relative, in % of the initial value) during degradation by (A) cellulases and (B) the cellulosome. Exemplary images from the data sets used to calculate the curves in panels A and B are shown in Fig. 4B and Fig. S13 (cellulases) and Fig. S17B (cellulosome). (C) Comparative analysis of degradation rates for the two systems, based on the data from panels A and B.

A

B

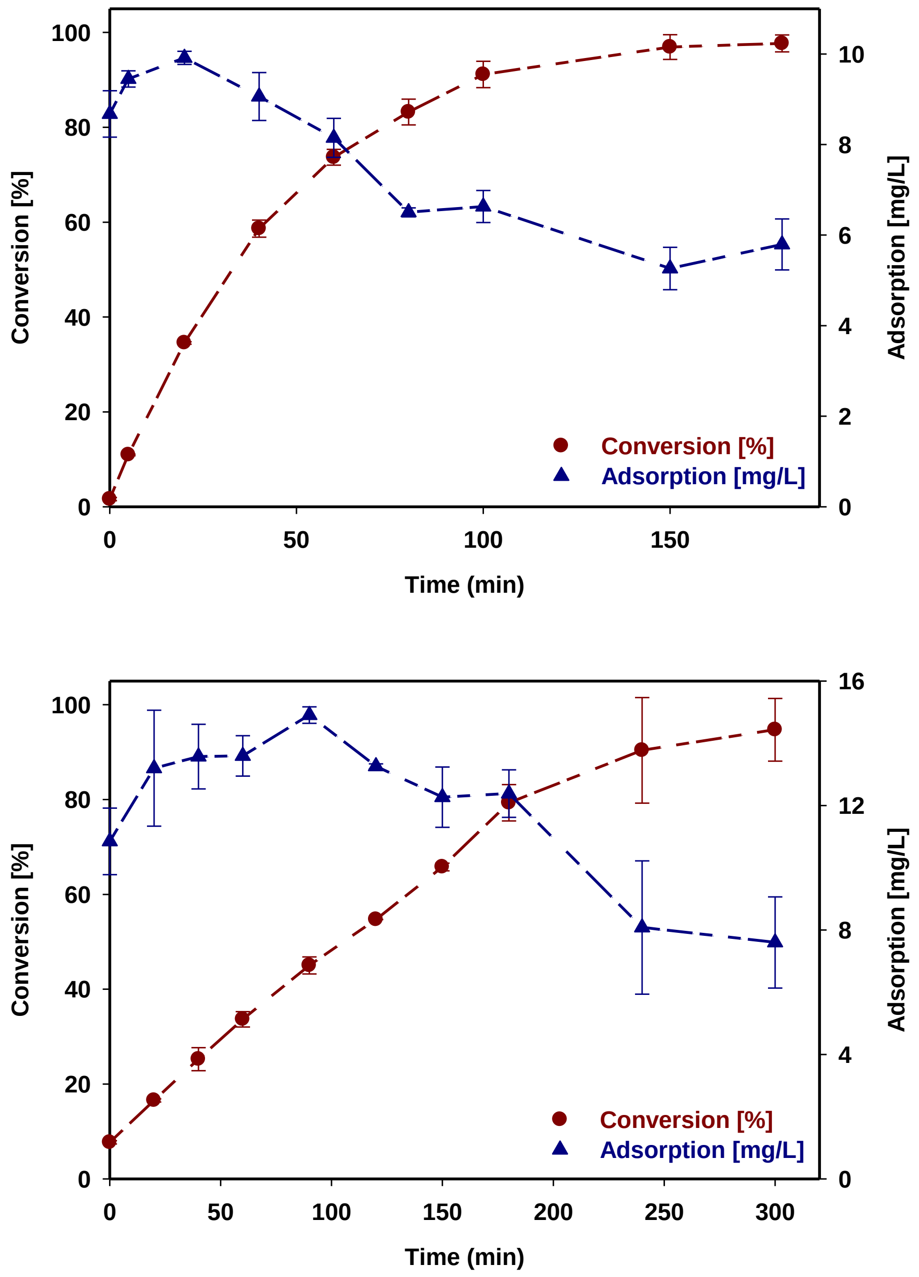

3.20 Fig. S20.

Cellulose conversion performed at elevated E/S ratios to monitor the temporal evolution of free enzyme in the solution. The figure shows cellulose conversion and simultaneous protein adsorption in (A) cellulases, and (B) cellulosome. Reactions for cellulases were carried out using 1.9 g/L BC and 17 mg/L enzyme. Reactions for cellulosome were carried out using 1.65 g/L BC and 20 mg/L enzyme. Error bars show the S.D. from triplicate reactions. The data were used to calculated the values of R_{ads} in Fig. 6A of main text.

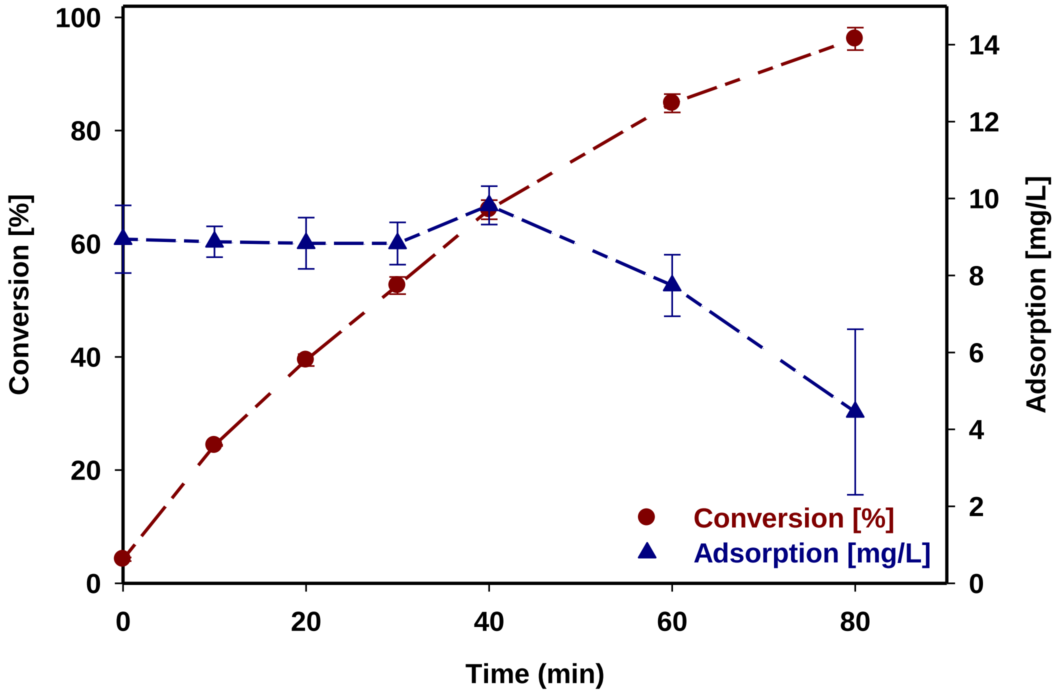

3.21 Fig. S21.

Reactivity and adsorption of cellulases on partially hydrolyzed BC (~52%). The original BC was hydrolyzed by cellulases and used for another round of reaction (see the section 2.1). The figure shows cellulose conversion and simultaneous protein adsorption by the cellulases on the partially hydrolyzed BC. To reproduce the E/S ratio at ~50% conversion in the original reaction, the measurements were performed by adding fresh cellulases (17 mg/L) to 1.0 g/L of partially hydrolyzed BC. Error bars show the S.D. from triplicate reactions. The data were used to calculated the values of R_{ads} in Fig. 6B (upper panel) of main text.

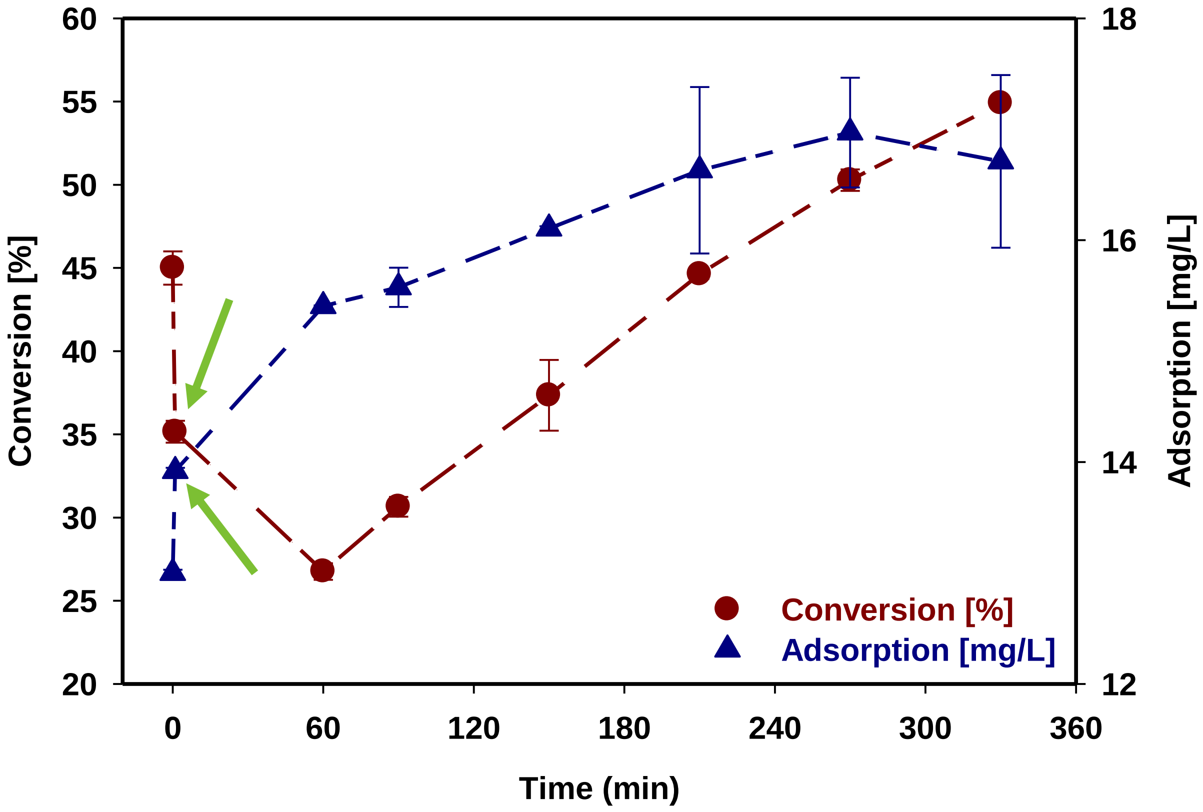

3.22 Fig. S22.

Experiment to assess the role of free enzyme in supernatant on the slowdown of BC hydrolysis by the cellulosome. The experiment was designed in two steps. First, a reaction of 20 mg/L cellulosome and 1.65 g/L BC was performed. The E/S ratio (= 12 mg/g) was chosen high to ensure that a substantial portion of the used enzyme remained unadsorbed. After 100 min, the whole reaction mixture was transferred to another tube containing BC pellet in an amount corresponding to additional 2.3 g/L. The mixture was agitated gently with a pipette tip (1 min) to resuspend the pelleted BC fully in the liquid. The BC addition decreased the E/S ratio to 2.7 which based on adsorption experiments largely depletes free enzyme from solution. The total volume change was small (≤ 4%). Cellulose conversion and protein adsorption were measured and are shown in the graph. The arrows indicate the corresponding data points after BC addition. Error bars show the S.D. from triplicate reactions. The data were used to calculated the values of R_{ads} in Fig. 6B (lower panel) of main text.

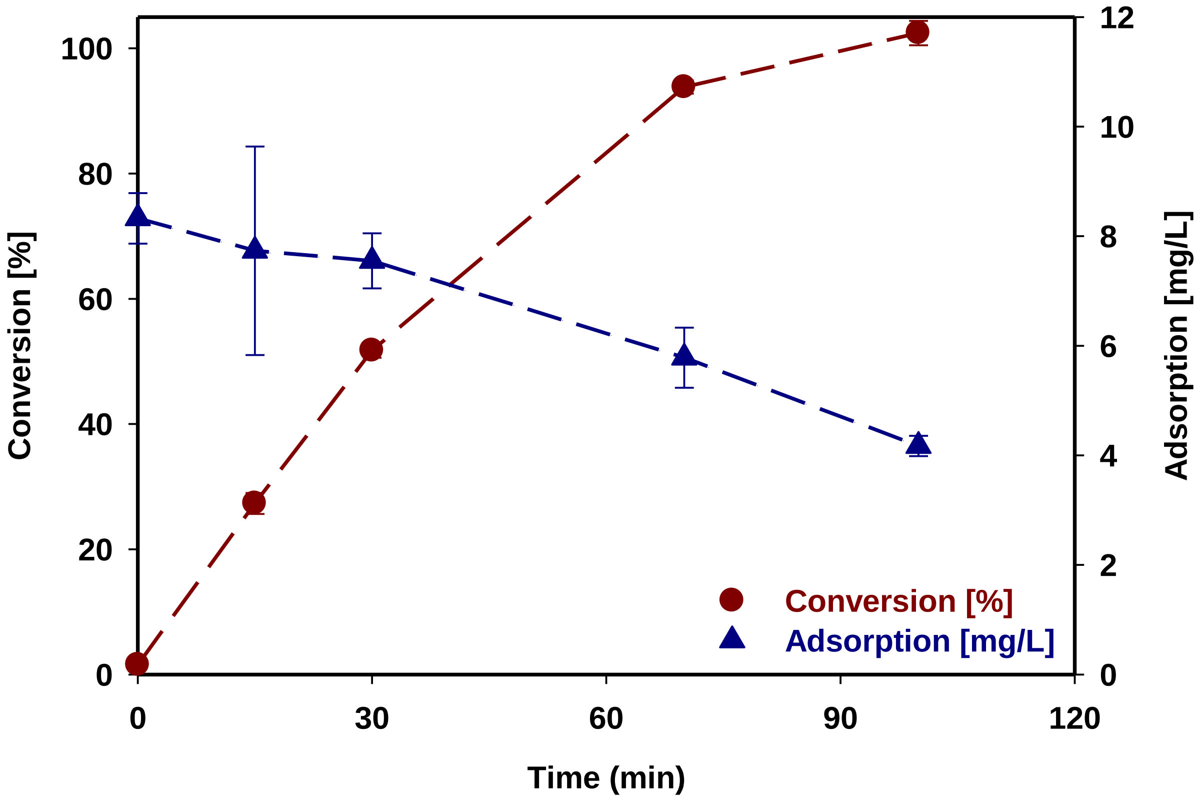

3.23 Fig. S23.

Reactivity and adsorption of cellulases on partially hydrolyzed BC (~45%), generated by pretreatment with the cellulosome. For further details on cellulosome pretreatment please refer to the section 2.1. The figure shows cellulose conversion and simultaneous protein adsorption by cellulases on partially hydrolyzed BC. To reproduce the E/S ratio at ~50% conversion in the original reaction, the measurements were performed by adding fresh cellulases (17 mg/L) to 1.0 g/L of partially hydrolyzed BC. Error bars show the S.D. from triplicate reactions. The data were used to calculated the values of R_{ads} in Fig. 6C (dark green symbols) of main text.

3.24 Table S1. Reaction conditions for conversion of different substrates shown in Fig. S1.

Enzyme and substrate	E/S (mg/g)	CI*	Ref.
T. reesei cellulases mix and BC	2	85-91	n.a.[#]
Cellic® CTec 3 HS and pulp	10	75	[21]
T. reesei cellulases mix and Avicel	3.6	91	[9]
Commercial *T. reesei* cellulases (Celluclast) and corn stover	5.5	50	[22]

*CI (crystallinity index) values are based on Segal method.[23]

[#]Data was obtained in this work

3.25 Table S2. Compilation of mean and standard deviation of exemplary *E** datasets from elementary units

	Mean (MPa)	Std
Ex. EU 1	19.96	1.29
Ex. EU 2	19.31	2.37
Ex. EU 3	20.39	1.88
Ex. EU 4	19.77	1.68
Ex. EU 5	16.78	3.03
Ex. EU 6	19.94	2.16

4 Supporting Movie captions

4.1 Movie S1.

Time lapse observation of cellulases degrading a partial elementary unit. The experiment was conducted at room temperature and started by the addition of 10 µL buffer solution containing cellulases (20 µg/mL). Originally an area of 1000 × 333 nm was scanned with about 0.3 fpm. The step size was 2 nm/pixel. Scale bar, time stamps and false color scale are included in the video.

4.2 Movie S2.

Cellulases at high loading degrading an elementary unit. The experiment was conducted at room temperature and the degradation was initiated by the addition of 20 µL buffer solution containing cellulases (200 µg/mL). Snapshots of the video are provided in Fig. 3A. An area of about 250 × 250 nm is shown with about 0.5 fpm. The step size was 4 nm/pixel. Scale bar, time stamps and false color scale are included in the video. Data shown was used to calculate Fig. 4A and C.

4.3 Movie S3.

Time lapse observation of cellulases degrading an elementary unit at elevated temperature. The experiment was conducted at 35°C and started by the addition of 10 µL buffer solution containing cellulases (100 µg/mL). An area of 320 × 280 nm was scanned with about 0.4 fpm. The step size was 2 nm/pixel. Scale bar, time stamps and false color scale are included in the video. The data shown were used to calculate the time courses presented in Fig. S18A and S18C, as well as for additional analyses described therein.

4.4 Movie S4.

Time lapse observation of cellulosome degrading multiple elementary units of a BC fiber. The experiment was done at 35°C and started by the addition of 10 µL buffer solution containing cellulosomes (100 µg/mL). Snapshots of the movie and a description of the degradation mode is provided Fig. 3C-E and the corresponding caption. An area of about 1000 × 340 nm is shown with about 0.5 fpm. The step size was 2 nm/pixel. Scale bar, time stamps and false color scale are included in the video. Data shown was used to calculate Fig. 4B and C.

4.5 Movie S5.

Time lapse observation of cellulosome degrading an elementary unit of a BC fiber. The experiment was done at 35°C using a setup as described for Movie S4. Scale bar, time stamps and false color scale are included in the video. Data shown was used to calculate Fig. S18B and C.

5 Supporting References

(1) Esterbauer, H.; Steiner, W.; Labudova, I.; Hermann, A.; Hayna, M. Production of *Trichoderma* Cellulase in Laboratory and Pilot Scale. *Bioresour. Technol.* 1991, *36* (1), 51–65. https://doi.org/https://doi.org/10.1016/0960-8524(91)90099-6.

(2) Zajki-Zechmeister, K.; Kaira, G. S.; Eibinger, M.; Seelich, K.; Nidetzky, B. Processive Enzymes Kept on a Leash: How Cellulase Activity in Multienzyme Complexes Directs Nanoscale Deconstruction of Cellulose. *ACS Catal.* 2021, *11* (21), 13530–13542. https://doi.org/10.1021/acscatal.1c03465.

(3) Mohammadkazemi, F.; Azin, M.; Ashori, A. Production of Bacterial Cellulose Using Different Carbon Sources and Culture Media. *Carbohydr. Polym.* 2015, *117*, 518–523. https://doi.org/10.1016/j.carbpol.2014.10.008.

(4) Jalak, J.; Väljamäe, P. Multi-Mode Binding of Cellobiohydrolase Cel7A from *Trichoderma Reesei* to Cellulose. *PLoS One* 2014, *9* (9), e108181. https://doi.org/10.1371/journal.pone.0108181.

(5) Mudinoor, A. R.; Goodwin, P. M.; Rao, R. U.; Karuna, N.; Hitomi, A.; Nill, J.; Jeoh, T. Interfacial Molecular Interactions of Cellobiohydrolase Cel7A and Its Variants on Cellulose. *Biotechnol. Biofuels* 2020, *13* (1), 1–16. https://doi.org/10.1186/s13068-020-1649-7.

(6) Cruys-Bagger, N.; Elmerdahl, J.; Praestgaard, E.; Borch, K.; Westh, P. A Steady-state Theory for Processive Cellulases. *FEBS J.* 2013, *280* (16), 3952–3961. https://doi.org/10.1111/febs.12397.

(7) Payne, C. M.; Knott, B. C.; Mayes, H. B.; Hansson, H.; Himmel, M. E.; Sandgren, M.; Ståhlberg, J.; Beckham, G. T. Fungal Cellulases. *Chem. Rev.* 2015, *115* (3), 1308–1448. https://doi.org/10.1021/cr500351c.

(8) Jalak, J.; Kurašin, M.; Teugjas, H.; Väljamäe, P. Endo-Exo Synergism in Cellulose Hydrolysis Revisited. *J. Biol. Chem.* 2012, *287* (34), 28802–28815. https://doi.org/10.1074/jbc.M112.381624.

(9) Eibinger, M.; Bubner, P.; Ganner, T.; Plank, H.; Nidetzky, B. Surface Structural Dynamics of Enzymatic Cellulose Degradation, Revealed by Combined Kinetic and Atomic Force Microscopy Studies. *FEBS J.* 2014, *281* (1), 275–290. https://doi.org/10.1111/febs.12594.

(10) Zajki-Zechmeister, K.; Eibinger, M.; Nidetzky, B. Enzyme Synergy in Transient Clusters of Endo- and Exocellulase Enables a Multilayer Mode of Processive Depolymerization of Cellulose. *ACS Catal.* 2022, *12* (17), 10984–10994. https://doi.org/10.1021/acscatal.2c02377.

(11) Nečas, D.; Klapetek, P. Gwyddion: An Open-Source Software for SPM Data Analysis. *Open Phys.* 2012, *10* (1), 181–188. https://doi.org/10.2478/s11534-011-0096-2.

(12) Guzman, H. V.; Perrino, A. P.; Garcia, R. Peak Forces in High-Resolution Imaging of Soft Matter in Liquid. *ACS Nano* 2013, *7* (4), 3198–3204. https://doi.org/10.1021/nn4012835.

(13) Nakamura, K.; Wada, M.; Kuga, S.; Okano, T. Poisson's Ratio of Cellulose I β and Cellulose II. *J. Polym. Sci. B Polym. Phys.* 2004, *42* (7), 1206–1211. https://doi.org/10.1002/polb.10771.

(14) Wu, X.; Moon, R. J.; Martini, A. Crystalline Cellulose Elastic Modulus Predicted by Atomistic Models of Uniform Deformation and Nanoscale Indentation. *Cellulose* 2013, *20* (1), 43–55. https://doi.org/10.1007/s10570-012-9823-0.

(15) Pittinger, B.; Erina, N.; Su, C. *Application Note #128 Quantitative Mechanical Property Mapping at the Nanoscale with PeakForce QNM*; 2012. https://www.bruker.com/en/products-and-solutions/microscopes/materials-afm/resource-library/an-128-quantitative-mechanical-property-mapping-at-the-nanoscale-with-peakforce-qnm.html?scrollToFormContent=embedform-85703228f5 (accessed 2025-09-02).

(16) Babi, M.; Williams, A.; Reid, M.; Grandfield, K.; Bassim, N. D.; Moran-Mirabal, J. M. Unraveling the Supramolecular Structure and Nanoscale Dislocations of Bacterial Cellulose Ribbons Using Correlative Super-Resolution Light and Electron Microscopy. *Biomacromolecules* 2023, *24* (1), 258–268. https://doi.org/10.1021/acs.biomac.2c01108.

(17) Cosgrove, D. J.; Dupree, P.; Gomez, E. D.; Haigler, C. H.; Kubicki, J. D.; Zimmer, J. How Many Glucan Chains Form Plant Cellulose Microfibrils? A Mini Review. *Biomacromolecules* 2024, *25* (10), 6357–6366. https://doi.org/10.1021/acs.biomac.4c00995.

(18) Li, L.; Zhang, P.; Li, J.; Wang, Y.; Wei, Y.; Hu, J.; Zhou, X.; Xu, B.; Li, B. Measurement of Nanomechanical Properties of DNA Molecules by PeakForce Atomic Force Microscopy Based on DNA Origami. *Nanoscale* 2019, *11* (11), 4707–4711. https://doi.org/10.1039/C8NR10354B.

(19) Artzi, L.; Bayer, E. A.; Moraïs, S. Cellulosomes: Bacterial Nanomachines for Dismantling Plant Polysaccharides. *Nat. Rev. Microbiol.* 2016, *15* (2), 83–95. https://doi.org/10.1038/nrmicro.2016.164.

(20) Hinterdorfer, P.; Dufrêne, Y. F. Detection and Localization of Single Molecular Recognition Events Using Atomic Force Microscopy. *Nat. Methods* 2006, *3* (5), 347–355. https://doi.org/10.1038/nmeth871.

(21) Wu, J.; Dong, Y.; Zhang, H.; Liu, J.; Renneckar, S.; Saddler, J. Reduced Cellulose Accessibility Slows down Enzyme-Mediated Hydrolysis of Cellulose. *Bioresour. Technol.* 2023, *371*, 128647. https://doi.org/10.1016/j.biortech.2023.128647.

(22) Yang, J.; Zhang, X.; Yong, Q.; Yu, S. Three-Stage Hydrolysis to Enhance Enzymatic Saccharification of Steam-Exploded Corn Stover. *Bioresour. Technol.* 2010, *101* (13), 4930–4935. https://doi.org/10.1016/j.biortech.2009.09.079.

(23) Segal, L.; Creely, J. J.; Martin, A. E.; Conrad, C. M. An Empirical Method for Estimating the Degree of Crystallinity of Native Cellulose Using the X-Ray Diffractometer. *Text. Res. J.* 1959, *29* (10), 786–794. https://doi.org/10.1177/004051755902901003.
